# FOXA1-driven transcription and TRIM9-mediated ubiquitination of LCN2 modulate ferroptosis in experimental sepsis-induced acute lung injury

**DOI:** 10.3389/fimmu.2026.1822078

**Published:** 2026-10-01

**Authors:** Jin Li, Yazhou Li, Chuntao Song, Jie Su, Lantao Wang, Shimin Dong

**Affiliations:** 1 Department of Emergency, The Fourth Hospital of Hebei Medical University, Shijiazhuang, Hebei, China; 2 Department of Emergency, Hebei Medical University Third Hospital, Shijiazhuang, Hebei, China

**Keywords:** ferroptosis, forkhead box protein A1, human pulmonary microvascular endothelial cells, lipocalin 2, lipopolysaccharide (LPS), sepsis-induced acute lung injury, tripartite motif-containing protein 9

## Abstract

**Background:**

Sepsis-induced acute lung injury (ALI) remains a devastating complication of sepsis, marked by uncontrolled pulmonary inflammation, alveolar-capillary barrier disruption, and high mortality. Ferroptosis, an iron-dependent form of regulated cell death driven by lipid peroxidation, is implicated in sepsis-related organ dysfunction. Moreover, a ferroptosis-related gene, Lipocalin 2 (LCN2), has been reported as a potential biomarker for sepsis-induced organ damage. However, the role and mechanism of LCN2 in sepsis-triggered ALI are still unclear.

**Methods:**

Transcriptomic data from GSE32707 were integrated for differential expression and WGCNA analysis. One overlapping gene was identified using key modules from WGCNA and ferroptosis-related genes from GeneCards database. LCN2 mRNA level was determined using real-time quantitative polymerase chain reaction (RT-qPCR). LCN2, Forkhead box protein A1 (FOXA1), tripartite motif-containing protein 9 (TRIM9), GPX4, SLC7A11, Occludin, and ZO-1 protein levels were detected using western blot. An *in vitro* model of endotoxemia was established in human pulmonary microvascular endothelial cells (HPMECs) by exposure to 10 µg/mL LPS for 24 h, recapitulating the inflammatory and endothelial injury responses relevant to sepsis-induced ALI. Cell viability and apoptosis were detected using CCK-8 and flow cytometry.

**Results:**

LCN2 was identified by intersecting WGCNA-derived MEsalmom and MEmidnightblue modules and ferroptosis-related genes from GeneCards database. LCN2 expression was upregulated in sepsis patients and LPS-treated HPMECs. LPS-induced HPMEC proliferation inhibition, and apoptosis, inflammatory response and ferroptosis promotion were partly abolished by LCN2 silencing. Mechanistically, FOXA1 activated LCN2 transcription by binding to its promoter region. TRIM9 promoted K48-linked polyubiquitination and subsequent degradation of LCN2. In CLP rats, LCN2 knockdown reduced lung injury and decreases pro-inflammatory cytokines, with accompanying changes in ferroptosis-related markers.

**Conclusion:**

This study demonstrates that FOXA1 transcriptionally activates LCN2, while TRIM9 promotes K48-linked ubiquitination and degradation of LCN2, together forming a dual-level regulatory mechanism that modulates ferroptosis and inflammation in LPS-treated HPMECs. Moreover, LCN2 knockdown alleviated lung injury with accompanying changes in ferroptosis-related markers in the CLP rat model, supporting the relevance of this axis *in vivo*. These findings identify LCN2 as a mechanistically relevant node in sepsis-induced ALI and provide a preclinical experimental basis for further validation.

## Introduction

As a life-threatening condition with a high mortality rate, sepsis is characterized by dysregulated host responses to infection, resulting in systemic inflammation and multi-organ failure (1–3). The lungs are the most vulnerable target organs during the course of sepsis. In general, excessive activation of inflammatory mediators could disrupt the body’s response to these substances, leading to a persistent positive feedback loop of amplification and destruction. Consequently, a cytokine storm is triggered that disrupts the structural integrity of the alveolar-capillary endothelial barrier, thereby exacerbating sepsis-induced acute lung injury (ALI) and ultimately progressing to acute respiratory distress syndrome (ARDS) (4). It has been reported that approximately 25%-50% of patients with sepsis could develop ALI or even ARDS, accounting for over 10% of all intensive care unit (ICU) admissions (5, 6). Despite substantial advances in various treatment options, such as antibiotic therapy, fluid resuscitation, immunomodulatory, cellular, and anti-inflammatory therapy, the treatment outcomes remain unsatisfactory (7). At present, the complex mechanisms underlying the development of sepsis-induced ALI remain unclear. Therefore, identifying suitable targets to mitigate tissue injury caused by sepsis holds potential clinical value.

Currently, ferroptosis has received increasing attention as a potential therapeutic strategy for the progression of sepsis-induced ALI (8). As an iron-dependent and non-apoptotic form of cell death, ferroptosis is mainly triggered by free iron and reactive oxygen species (ROS), thereby inactivating the antioxidant defense system in cells (9, 10). In general, the intracellular labile iron (Fe^2+^) is central to ferroptosis, which catalyzes the Fenton reaction to generate hydroxyl radicals, thereby initiating lipid peroxidation (11). Then, overwhelming membrane lipid peroxidation could compromise plasma membrane permeability and integrity, ultimately resulting in membrane rupture and cell death. Previous studies have indicated that the glutathione (GSH)-glutathione peroxidase 4 (GPX4) axis represents the primary defense against ferroptosis. As a key antioxidant enzyme, GPX4 could reduce lipid peroxides to their corresponding non-toxic alcohols by catalyzing the conversion of GSH (12), thereby preventing ferroptosis and sustaining cell viability. However, the significant decrease in GPX4 protein level during sepsis-induced ALI leads to an increased sensitivity of endothelial cells to ferroptosis, which subsequently causes vascular barrier disruption and secondary pulmonary edema (13). It has been reported that ferroptosis could amplify tissue injury by iron-dependent lipid peroxidation in alveolar epithelial cells during sepsis-induced ALI. Hence, ferroptosis has great research value in sepsis-induced ALI. As a secreted glycoprotein, Lipocalin 2 (LCN2) could induce the release of multiple chemokines involved in inflammatory responses, playing a pivotal role in diverse biological processes (14, 15). Early studies have found that LCN2 is strongly activated in inflamed organs, including the lungs, heart, liver, and kidney (16). Under multiple cellular stress conditions, LCN2 is consistently upregulated, serving as a key regulator for oxidative stress, iron metabolism, and inflammatory response in mammals (17, 18). Indeed, a recent study has suggested that LCN2 deficiency could alleviate sepsis-induced liver injury by repressing inflammation, oxidative stress, and organ injury via inhibition of ferroptosis (19). Nevertheless, no studies have investigated the changes in LCN2 during septic lung injury or whether LCN2 participates in the process of ferroptosis.

Increasing evidence has indicated that transcription factors are a category of proteins capable of regulating gene transcription, which could selectively recognize and bind to specific DNA sequences in the promoter regions, promoting or inhibiting gene expression (20). As an evolutionarily conserved winged helix transcription factor with various regulatory functions, forkhead box A 1 (FOXA1) could build gene expression capacity and partake in modulating the growth and upkeep of organ systems (21). Moreover, previous studies have indicated that the lack of FOXA1 could hinder oxidative stress, apoptosis, and endoplasmic reticulum stress (22, 23). Notably, it has been reported that FOXA1 knockdown could suppress inflammatory response, ROS production, and ferroptosis in lipopolysaccharide (LPS)-induced alveolar epithelial cells by regulating SLC7A11 transcription (24), suggesting that FOXA1 might play an important role in septic lung injury.

Ubiquitination is a key protein post-translational modification that influences the fate and function of proteins by labeling substrate proteins with ubiquitin (25, 26). This process involves marking proteins for degradation through the coordinated action of three key enzymes: ubiquitin-activating (E1), -conjugating (E2), or -ligating (E3) enzymes (27). Among them, tripartite motif (TRIM) family belongs to RING-finger-containing ligases, representing the largest protein group within E3 ubiquitin ligases (28). Previous studies have suggested that TRIM proteins could regulate ferroptosis and inflammation through ubiquitin-dependent pathways (29, 30). Of interest, tripartite motif-containing protein 9 (TRIM9) could suppress neuroinflammation by regulating the NF-κB pathway (31). Herein, this work found that LCN2 expression was increased in LPS-treated HPMECs and cecal ligation and puncture (CLP)-induced septic rats, and FOXA1 could sustain LCN2 expression by directly binding to its promoter region and activating transcription. Furthermore, TRIM9 was found to promote ubiquitination and destabilization of LCN2. Accordingly, this research aimed to investigate whether FOXA1-activated transcriptions and TRIM9-mediated ubiquitination and degradation of LCN2 could modulate sepsis-induced ALI through ferroptosis and inflammatory response.

## Materials and methods

### Data collection and processing

In this research, the microarray dataset GSE32707 was downloaded and collected from the Gene Expression Omnibus (GEO) repository. The data obtained based on the sequencing platform GPL10558, contained gene expression information of 144 whole blood samples, of which health controls and 31 sepsis-induced ALI were selected for inclusion in this study. Then, differential expression analysis was done using the “limma” R package with the Benjamini–Hochberg false discovery rate (FDR) correction for multiple comparisons. According to the selection criteria: |logFC| ≥2 and FDR < 0.05, DEGs were identified. The heatmap and volcano plot were carried out using the sangerbox platform (http://www.sangerbox.com/home.html).

### Analysis of gene co-expression networks using weights

To systematically excavate the key gene modules in the control group and sepsis-induced ALI, co-expression networks were constructed using WGCNA package in R. After removing outliers, the soft-thresholding power (β=16) and R^2^ = 0.885 were selected using the pickSoftThreshold function to guarantee a scale-free topology. Then, gene interconnectedness was assessed via the Topological Overlap Matrix (TOM), followed by module identification using dynamic tree cutting. ey parameters included the minimum module size (minModuleSize = 30), split depth control (deepSplit = 2), the PAM optimization stage (pamStage = TRUE), and the module merging heterogeneity threshold (mergeCutHeight = 0.25), ultimately yielding 16 modules. After calculating the module eigengene (ME) for each module, modules with p-values < 0.05 and |correlation coefficient| > 0.3 were defined as significantly correlated modules. For key modules, core genes (Hub genes) with both high module membership (MM/kME > 0.8) and high gene significance (GS > 0.2) were further selected for subsequent analysis. In addition, the WGCNA-derived MEsalmom and MEmidnightblue modules were intersected with ferroptosis-related genes from GeneCards database via Venn diagram analysis. It should be noted that hub genes—rather than all genes within the selected modules—were chosen for subsequent intersection analysis to focus on the most functionally important nodes within the co-expression network. This approach minimizes potential background interference from low-connectivity peripheral genes and ensures that the overlapping gene(s) identified are more likely to be biologically relevant to sepsis-induced ALI and ferroptosis.

### Cell culture

Human pulmonary microvascular endothelial cells (HPMECs, CP-H001, Procell, Wuhan, China) were routinely maintained in the specific medium (CM-H001, Procell) at 37˚C in a humidified 5% CO_2_ incubator. To mimic the inflammatory and endothelial injury responses relevant to endotoxemia *in vitro*, HPMECs were exposed to 10 µg/mL LPS (Sigma-Aldrich, St. Louis, MO, USA) for 24 h.

### Real-time quantitative polymerase chain reaction

In short, total RNA from tissues or cells was isolated using Trizol reagent (Invitrogen, Paisley, Scotland, UK), followed by the determination of RNA concentration and purity. Then, 1 µg RNA per sample was applied to synthesize cDNA product using HiScript 1st Strand cDNA synthesis kit (Vazyme, Nanjing, Shanghai). After that, amplification reaction was implemented on ABI7500 quantitative PCR system (Applied Biosystems, Warrington, UK) with PerfectStart Green qPCR SuperMix (TransGen Biotech, Beijing, China). After normalizing to glyceraldehyde-3-phosphate dehydrogenase (GAPDH), results were analyzed using the 2^–ΔΔCt^ method. Primer sequences are listed in **Table 1**.

**Table 1:** Primer sequences used for qPCR.

Gene	Direction	Primer sequence (5’-3’)
LCN2	Forward	ATTCTGCTGTCCCCATCTCG
	Reverse	CCAGCTCCCTCAATGGTGTT
GAPDH	Forward	AAGCCTGCCGGTGACTAAC
	Reverse	GCATCACCCGGAGGAGAAAT

### Western blot assay

In brief, the extraction of total protein was conducted using Radio Immunoprecipitation Assay (RIPA) buffer (Keygen, Nanjing, China), followed by quantification using BCA protein assay kit. Then, samples were subjected to 10% SDS polyacrylamide gel for separation and electro-transfer onto PVDF membranes. Then, blots were probed with primary antibody incubation at 4˚C: LCN2 (#44058, 1:1000, CST, Beverly, MA, USA), TRIM9 (#47990, 1:1000, CST), GPX4 (#52455, 1:1000, CST), SLC7A11 (#12691, 1:1000, CST), Occludin (13409-1-AP, 1:2000, proteintech, Wuhan, China), ZO-1 (21773-1-AP, 1:5000, proteintech), FOXA1 (PA5-27157, 1:1000, Invitrogen), and GAPDH (ab8245, 1:1000, Abcam, Cambridge, MA, USA) overnight. After washing, the membranes were incubated with secondary antibody, followed by the detection of blots using ECL reagent and ImageJ.

### Cycloheximide treatment

To assess the stability of LCN2 protein, HPMECs were transfected with OE-NC or OE-TRIM9, followed by treatment with Cycloheximide (CHX, 50 μg/mL, Sigma-Aldrich) for 0, 4, 8, and 12 h. Then, the collected cells were subjected to lysis and western blot analysis.

### Cell transfection

Briefly, small interference RNA (siRNAs) targeting LCN2 (si-LCN2 and si-LCN2#2), FOXA1 (si-FOXA1), TRIM9 (si-TRIM9), and their negative control (si-NC) were acquired from RiboBio (Guangzhou, China). Furthermore, the cDNA sequence of human LCN2 or human TRIM9 was synthesized and inserted into pcDNA 3.1 vector (GenePharma, Shanghai, China), generating pcDNA 3.1-LCN2 (OE-LCN2) or pcDNA 3.1-TRIM9 (OE-TRIM9). Meanwhile, pcDNA empty vector acted as a negative control (OE-NC). Finally, transfection of oligonucleotides (20 nM) and plasmids (50 ng) was performed with Lipofectamine 3000 reagent (Invitrogen) for 48 h, followed by LPS treatment.

### Cell counting kit-8 assay

Cell viability was detected using CCK-8 reagent (Beyotime). After various treatments, 1×10^4^ HPMECs in 96-well plates were incubated with 10 μL of CCK-8 reagent for 2 h at 37˚C. Finally, cell viability was evaluated by recording absorbance at 450 nm using a microplate reader.

### Cell apoptosis assay

In short, the harvested HPMECs were washed twice with pre-cooled PBS, followed by re-suspending in binding buffer (Solarbio, Beijing, China). Then, 5 μL Annexin V-FITC (Solarbio) was added to the cell suspension, which was stained with 10 μL PI solution (Solarbio) for 10 min in the dark. Finally, apoptotic cells were analyzed using a flow cytometer.

### ELISA

The levels of Tumor necrosis factor α (TNF-α), interleukin-1β (IL-1β), and IL-10 levels in tissue homogenates or HPMEC culture supernatant were detected using Rat/human TNF-α ELISA kit (PT516/PT518, Beyotime), Rat/human IL-1β ELISA kit (PI303/PI305, Beyotime), and Rat/human IL-10 ELISA kit (PI525/PI528, Beyotime). Finally, results were analyzed using a microplate reader at A450 nm.

### Assessment of intracellular malondialdehyde, glutathione, and Fe^2+^ levels

For MDA and GSH measurement, HPMECs and tissues were harvested and lysed. Subsequently, MDA Kit (S0131S, Beyotime) and GSH kit (CS0260, Sigma-Aldrich) were applied to determine the MDA and GSH levels in cell and tissues extracts.

For intracellular ferrous iron (Fe^2+^) content, the collected HPMECs and tissues were washed with cold PBS and homogenized in iron assay buffer. Then, Iron colorimetric assay kit (E1042, E1050, Applygen, Beijing, China) and a microplate reader were utilized to determine the Fe^2+^ level in the collected cell and tissue supernatant.

### Detection of reactive oxygen species

Lipid peroxidation was detected using a C11-BODIPY fluorescent probe (Invitrogen). Briefly, 1×10^5^ HPMECs in 6-well plates were cultured overnight. After treatment, cells were labeled with 5 μM C11-BODIPY in Hank’s balanced salt solution at 37˚C. 30 min later, a flow cytometer was used to assess the fluorescence intensity (lipid-ROS level) in the harvested cells.

### Chromatin immunoprecipitation

After JASPAR prediction, the possible FOXA1 binding sites in the LCN2 promoter were found, followed by verification using ChIP assay. In short, 1×10^6^ HPMECs were treated with 1% formaldehyde to cross-link proteins to DNA. Following glycine termination of the reaction, the cross-linked chromatin was lysed into 200-400 bp fragments. After digestion, anti-FOXA1 or anti-IgG was incubated with the chromatin fragment, followed by mixture with magnetic beads. At last, DNA was extracted from eluted chromatin for RT-qPCR analysis. Primer sequences were shown as follows: Forward: 5’-CTCCTGGGAGGGCCATGATT-3’, Reverse: 5’-TTTAAGGGTCAGGGAAGGGTG-3’.

### Dual-luciferase reporter assay

To assess the interaction between FOXA1 and the LCN2 promoter, this assay was performed in HPMECs. Briefly, wild-type (WT) LCN2 (5’-CGCCTTGTTTTTCCTTGTCCA-3’) or MUT LCN2 (5’-CGCCTCACCCCGAAGGGTCCA-3’) plasmid located on (-1403 ~ -1395 bp) was created by cloning the LCN2 harboring wild-type or mutant-type FOXA1 binding sites into psiCHECK2 vector. Then, HPMECs at sub-confluent levels were transfected with Lipofectamine Plus (Invitrogen) with the indicated plasmids and si-NC or si-FOXA1. After a 48-h period, the luciferase activities in cell lysates were analyzed by a Dual Luciferase Assay Kit (Promega, Madison, WI, USA).

### Co-immunoprecipitation assay

Interaction between TRIM9 and LCN2 was assessed in this assay. After being suspended in IP Lysis buffer (Invitrogen) for 15 min at 4 °C, the whole HPMEC lysates were generated and centrifuged. After that, the collected supernatants were incubated with anti-TRIM9, anti-LCN2, and anti-IgG for 16 h, followed by addition of protein-A/G magnetic beads (Millipore, Molsheim, France) for 12 h. After IP lysis buffer washing, the generated precipitates were determined by western blot.

### Ubiquitylation assay

In short, plasmids encoding Ub (wild-type K48 and K63 Ub plasmids) were co-transfected into HPMECs with or without OE-TRIM9 or Flag-LCN2, and then ubiquitination assay was performed using denatured IP (32). First of all, the cells were harvested with EBC buffer (MedChemExpress, Monmouth Junction, NJ, USA) containing 4% SDS and boiled at 95˚C for 15 min. After sonicating and centrifuging, 5% of the total lysate was reserved as input and subjected to Western blot analysis for LCN2, TRIM9, and GAPDH to verify transfection efficiency and equal loading. The remaining supernatant was diluted to one-tenth of the original concentration with SDS-free EBC buffer, followed by immunoprecipitation (IP) using anti-LCN2 antibody (Abcam) or species-matched normal rabbit IgG (CST) as a negative control, coupled to protein A/G magnetic beads (Millipore) overnight at 4˚C. After washing, the precipitated proteins were eluted by boiling in 2× SDS loading buffer. The eluates were resolved by SDS-PAGE and immunoblotted with anti-ubiquitin (#3936, 1:1,000, CST), anti-K48-linked ubiquitin (#8081, 1:1,000, CST), or anti-K63-linked ubiquitin (#5621, 1:1,000, CST) antibodies.

### Animal model

All procedures involved in this experiment were performed following the ARRIVE guidelines and received approval from the Animal Ethics Committee of the Fourth Hospital of Hebei Medical University (No. IACUC-4th Hos Hebmu-2024781). The sample size (n = 5 per group) was determined by power analysis using G*Power software (version 3.1). Exclusion criteria were predefined as follows: (1) rats that died before the scheduled endpoint (12 h post-CLP) due to non-experimental causes; (2) rats with evidence of cecal perforation not induced by the standardized puncture procedure; (3) rats showing signs of peritonitis prior to the scheduled endpoint. No animals met the exclusion criteria in this study. Twenty male Sprague-Dawley rats (6-8 weeks, SPF Biotech, Beijing, China) were housed in a specific pathogen-free condition. These rats were fed for 7 days under stable environmental temperature and lighting conditions. According to a computer-generated random number table, all rats were randomly divided into four groups (n=5 per group): 1) the cecal ligation and puncture (CLP) group: the rats were anesthetized with an intraperitoneal injection of sodium pentobarbital (30 mg/kg), after which their cecum was exposed and ligated 7.5 mm from its distal end. Then, the ligated cecum (approximately 1.5 mm) was punctured through and through for 4 times with a 21-gauge needle. After squeezing out the cecal contents, reposition the cecum and suture the incision at both the muscle and skin layers. Finally, rats received a subcutaneous injection of 37 °C saline solution (1 mL/100 g) and were returned to their cages for rewarming for 1 h; 2) the Sham group: rats only received exposure of the cecum without ligation or puncture; 3) the CLP+Ad-shNC group: 7 days before CLP induction, rats received a tail vein injected of 100 μL of adenovirus (Ad-NC, titer: 1 × 10¹² PFU/mL (HanBio, Shanghai, China); 4) the CLP+Ad-sh-LCN2 group: 7 days before CLP induction, rats received a tail vein injected of 100 μL of adenovirus (Ad-sh-LCN2, titer: 1 × 10¹² PFU/mL (HanBio). Virus administration was performed 7 days prior to CLP surgery to ensure adequate gene knockdown. All rats were paralyzed with continuous administration of 0.5 μg/g/h pentobarbital sodium (#P3761, Sigma-Aldrich) and muscle relaxation was achieved using 0.5 mg/kg cisatracurium once an hour. Animals were euthanized with 100-200 mg/kg pentobarbital sodium at 12 h after post-CLP (specifically, 12 h after the completion of the CLP surgical procedure). This time point was selected to capture the acute inflammatory and early fibroproliferative phase of sepsis-induced lung injury. Model establishment was considered successful when the following criteria were met: (1) the presence of lethargy, piloerection, and decreased activity; (2) a sustained decrease in mean arterial pressure (MAP) by ≥ 20% from baseline; and (3) histologically confirmed acute lung injury characterized by alveolar septal thickening, inflammatory cell infiltration, and hyaline membrane formation observed in HE-stained lung sections from the CLP group compared to the Sham group. All surviving rats met these criteria at the 12-h endpoint. Then, lung tissues were removed for follow-up research.

### Pathological examination of lung tissues

For hematoxylin and eosin (HE) staining, the collected rat lung tissues were fixed in 4% paraformaldehyde, embedded in paraffin, and cut into 4 μm sections. After staining with hematoxylin and eosin, the morphology was analyzed using a microscope. For Masson staining, paraffin-embedded lung tissues (4 μm) were stained with Masson trichrome staining kit (C0189S, Beyotime). Pathological evaluation of the lungs was performed by light microscopy based on an assessment of interstitial infiltrates, peribronchial and perivascular inflammation, alveolar infiltrates, alveolar hemorrhage and hyaline membranes, as determined by an independent pathologist who was blinded to the experimental groupings. All histological slides were coded with random numbers prior to evaluation, and the code was not broken until the scoring was completed. Lung injury was scored on a 5-point scale, as described previously (33).

### Lung wet/dry weight ratio

The W/D ratio of lungs was an indicator of pulmonary edema severity in rats. In short, the rat lung tissues were blotted dry and immediately weighed as wet weight. Then, lung tissues were placed in an oven at 80 °C for 72 h to obtain the dry weight. Finally, the Wet-to-dry weight ratio was calculated to evaluate pulmonary edema.

### Statistical analysis

All experiments were performed with at least three independent biological replicates, unless otherwise stated. *In vitro* assays were conducted with three biological replicates, each containing three technical replicates. GraphPad Prism7 was applied for statistical analysis. Measurement data were represented as mean ± standard deviation (SD). Data distribution was tested with Shapiro-Wilk test. Significant differences were assessed based on Student’s *t*-test or one-way ANOVA with Tukey’s tests. Significance threshold was set at a *P*-value < 0.05.

## Results

### Transcriptomic analysis identifies DEGs associated with sepsis-induced ALI

To explore efficient markers for diagnosis and treatment in sepsis-induced ALI patients, a microarray raw dataset GSE32707, containing healthy controls and 31 sepsis-induced ALI patients, was included as the training set. Subsequently, DEGs were identified by using limma package according to P-value < 0.05 and |LogFC|> 2. Then, Volcano plot showed a total of 692 DEGs, including 262 upregulated genes and 430 downregulated genes (**Figure 1A**), with red indicating upregulated genes and blue indicating downregulated genes. Moreover, the top 20 DEGs from the upregulated and downregulated genes were selected, respectively, for the Heatmap (**Figure 1B**).

**Figure 1:**
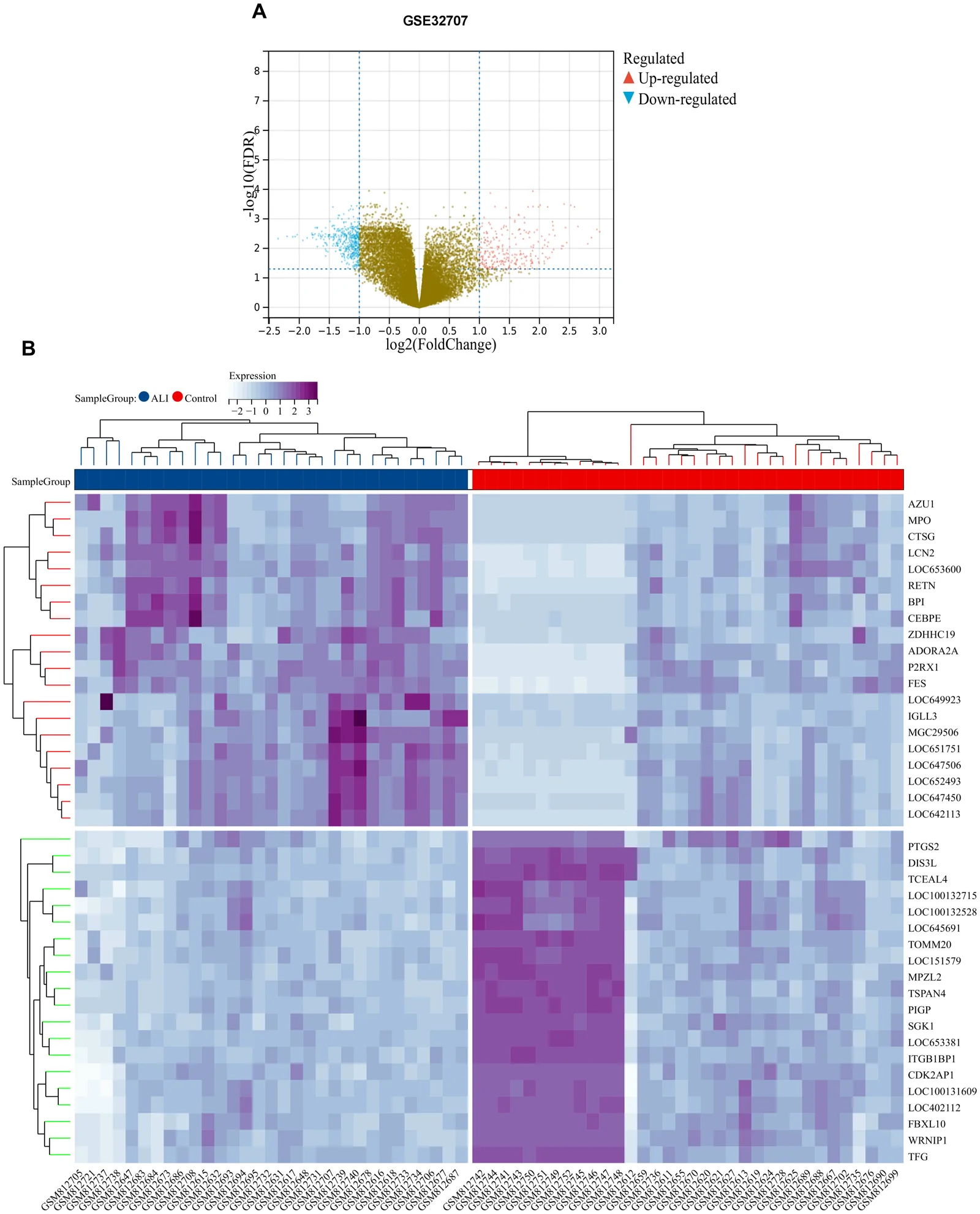
Analysis of differentially expressed genes (DEGs) in sepsis-induced acute lung injury (sepsis-induced ALI). **(A)** Volcano plot depicting the distribution of DEGs (red represent: 262 upregulated genes and blue represent: 430 downregulated genes) in sepsis samples versus sepsis-induced ALI samples from the GSE32707 database. **(B)** Heatmap illustrating the expression profiles of TOP 20 DEGs (upregulated and downregulated).

### Integrated bioinformatics analysis identifies LCN2 as a ferroptosis-related gene in sepsis-induced ALI

To identify gene modules with extensive co-expression patterns, WGCNA algorithm was utilized to analyze the GSE32707 dataset. Then, a soft-thresholding power of 16 was selected to achieve a scale-free topology of the network, where the corresponding R^2^ was 0.885, and the average connectivity was high (**Figures 2A, B**). Subsequently, hierarchical clustering combined with a dynamic tree-cutting algorithm identified 16 distinct co-expression modules (**Figure 2C**). Among them, MEsalmom module (R = 0.56, P = 1e−06) and MEmidnightblue module (R = 0.57, P = 6e−07) demonstrated the strongest correlation with sepsis-induced ALI. Based on this, 38 genes from the MEsalmome and MEmidnightblue modules were chosen for further analysis. Emerging evidence has indicated that ferroptosis plays a pivotal role in the progression of sepsis-induced ALI. Thus, ferroptosis-related genes implicated in ALI-induced sepsis were further identified. As shown in **Figure 2D**, the intersection of hub genes from two modules with 243 ferroptosis genes in the GeneCards database yielded one overlapping gene, LCN2. The identification of LCN2 as the sole overlapping gene highlights its unique and specific involvement in bridging ALI-associated core genes with the ferroptosis pathway, rather than reflecting an overly restrictive filtering approach. Together, these findings suggested that LCN2 might be involved in the regulation of ferroptosis in sepsis-induced ALI.

**Figure 2:**
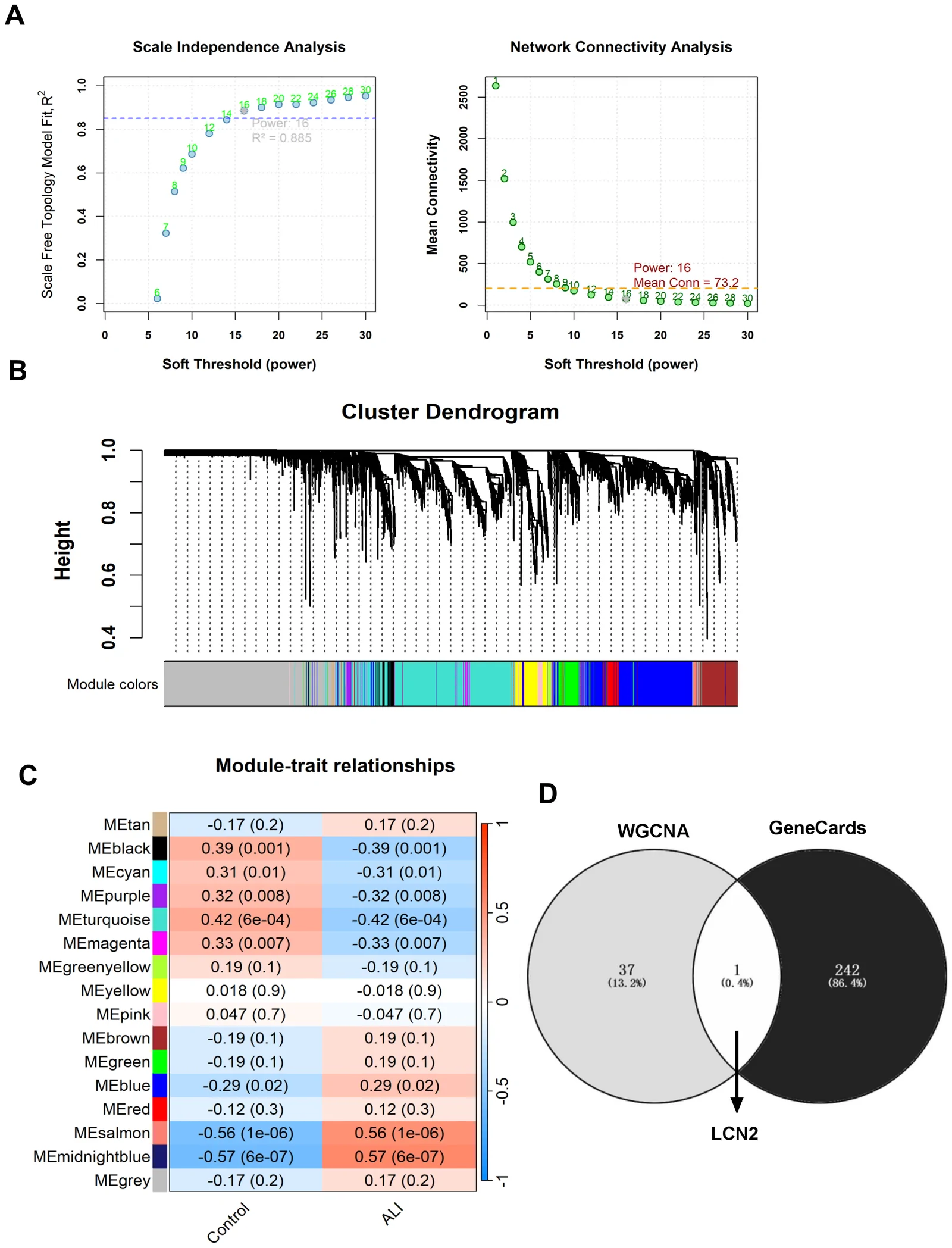
Construction of WGCNA and identification of key modules. **(A)** Analysis of the scale-free index and mean connectivity for various soft-thresholding powers (β). A soft-thresholding power = 16 was selected to ensure a scale-free network. **(B)** WGCNA cluster dendrogram and module assignment. The branches refer to clusters of genes that are highly connected. The colors in the horizontal bar represent the modules. **(C)** The correlation between module eigengenes and sepsis-induced ALI is depicted as a Heatmap, with each row showing the correlation and P-value. **(D)** Venn diagram showing 1 overlapping gene (LCN2) between WGCNA modules and GeneCards database.

### LCN2 is upregulated in sepsis patients and LPS-treated HPMECs

Next, to further evaluate the role of LCN2 in sepsis-induced ALI, its expression patterns were detected. Based on GSE32707 database analysis, LCN2 expression was markedly increased in 31 sepsis-induced ALI patients relative to 34 control samples (**Figure 3A**). Then, this study stimulated human pulmonary microvascular endothelial cells (HPMECs) using 10 µg/mL LPS for 24 h to mimic endotoxemia-related endothelial injury *in vitro*. After that, RT-qPCR and western blot results displayed that LCN2 mRNA level and protein level were obviously elevated in LPS-treated HPMECs in comparison with the Control group (**Figures 3B, C**).

**Figure 3:**
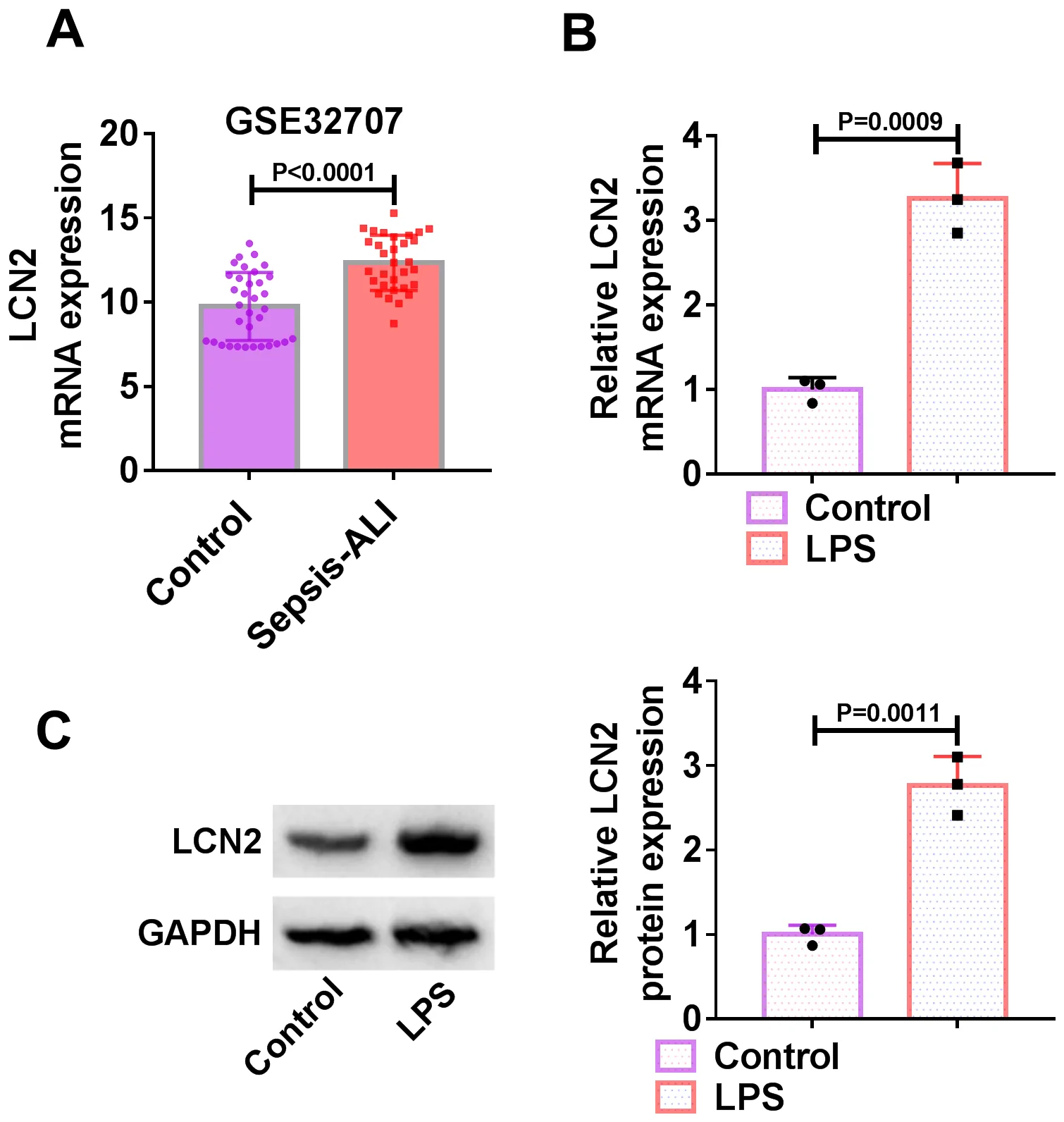
Expression patterns of LCN2 in LPS-treated HPMECs. **(A)** The GSE32707 database presented LCN2 expression in 31 sepsis samples and 34 sepsis-induced ALI samples. **(B, C)** LCN2 mRNA level and protein level were determined using RT-qPCR and western blot in HPMECs treated with 10 µg/mL LPS for 24 h or without (Control). Unpaired *t*-test, n=3.

### LCN2 silencing alleviates LPS-induced endothelial dysfunction and ferroptosis *in vitro*

Subsequently, to further identify the functional effects of LCN2 in LPS-treated HPMECs, loss-of-function analyses were performed *in vitro*. At first, western blot results showed that the LCN2 protein level was markedly reduced in si-LCN2-transfected HPMECs compared with the si-NC group (**Figure 4A**), implying that knockdown efficiency was available. After that, CCK-8 and flow cytometry analysis displayed that LPS stimulation could prominently impair HPMEC viability and promote cell apoptosis, which were partly abolished by LCN2 downregulation (**Figures 4B, C**). Furthermore, the secretion of pro-inflammatory cytokines TNF-α and IL-1β was highly induced, and anti-inflammatory cytokine IL-10 was obviously reduced in response to LPS treatment in HPMECs, which were abolished after si-LCN2 addition (**Figures 4D–F**). In terms of ferroptosis, the effects of LCN2 knockdown on the characteristic indicators of ferroptosis (MDA, Fe^2+^ level, Lipid-ROS, and GSH) in LPS-treated HPMECs were further assessed. In terms of ferroptosis, LPS treatment evidently increased MDA level, Fe^2+^ level, and Lipid-ROS production in HPMECs, whereas these influences were partially weakened by LCN2 interference (**Figures 4G, I**). In addition, the repression of LPS exposure on GSH level in HPMECs was ameliorated by LCN2 downregulation (**Figure 4J**). It has been reported that SLC7A11/GPX4 axis acts as the primary defense mechanism against ferroptosis due to its potent function to detoxify excess lipid peroxides with the help of GSH. Thus, the GPX4 and SLC7A11 protein levels were further determined. As shown in **Figure 4K**, western blot results displayed that LCN2 deficiency overturned LPS treatment-mediated GPX4 and SLC7A11 inhibition. In addition, the effects of LCN2, Ferrostatin-1 (ferroptosis inhibitor), and ferroptosis activator (Erastin) on cell viability were assessed in LPS-treated HPMECs. As shown in **Supplementary Figure S1**, Ferrostatin-1 (ferroptosis inhibitor) relieved the repression of LCN2 overexpression on cell viability in LPS-treated HPMECs, and ferroptosis activator (Erastin) abolished the promotion role of LCN2 silencing on cell viability in LPS-treated HPMECs. These results suggested that LCN2 regulated LPS-mediated HPMEC viability via ferroptosis. Previous studies have suggested that Occludin and ZO-1 are essential tight junction proteins that maintain epithelial and endothelial barrier integrity by linking transmembrane proteins to the cytoskeleton and regulating paracellular permeability. To further evaluate the effects of LCN2 deficiency on endothelial barrier function, Occludin and ZO-1 expression levels were measured using western blot assay. Data showed that LPS treatment-triggered Occludin and ZO-1 repression in HPMECs were partly relieved by LCN2 knockdown (**Supplementary Figure S2**). In addition, si-LCN2#2 transfection could relieve LPS-triggered endothelial dysfunction and ferroptosis (**Supplementary Figure S3**). Collectively, these data suggested that LCN2 silencing could attenuate LPS-induced endothelial dysfunction by inhibiting inflammation and ferroptosis *in vitro.*

**Figure 4:**
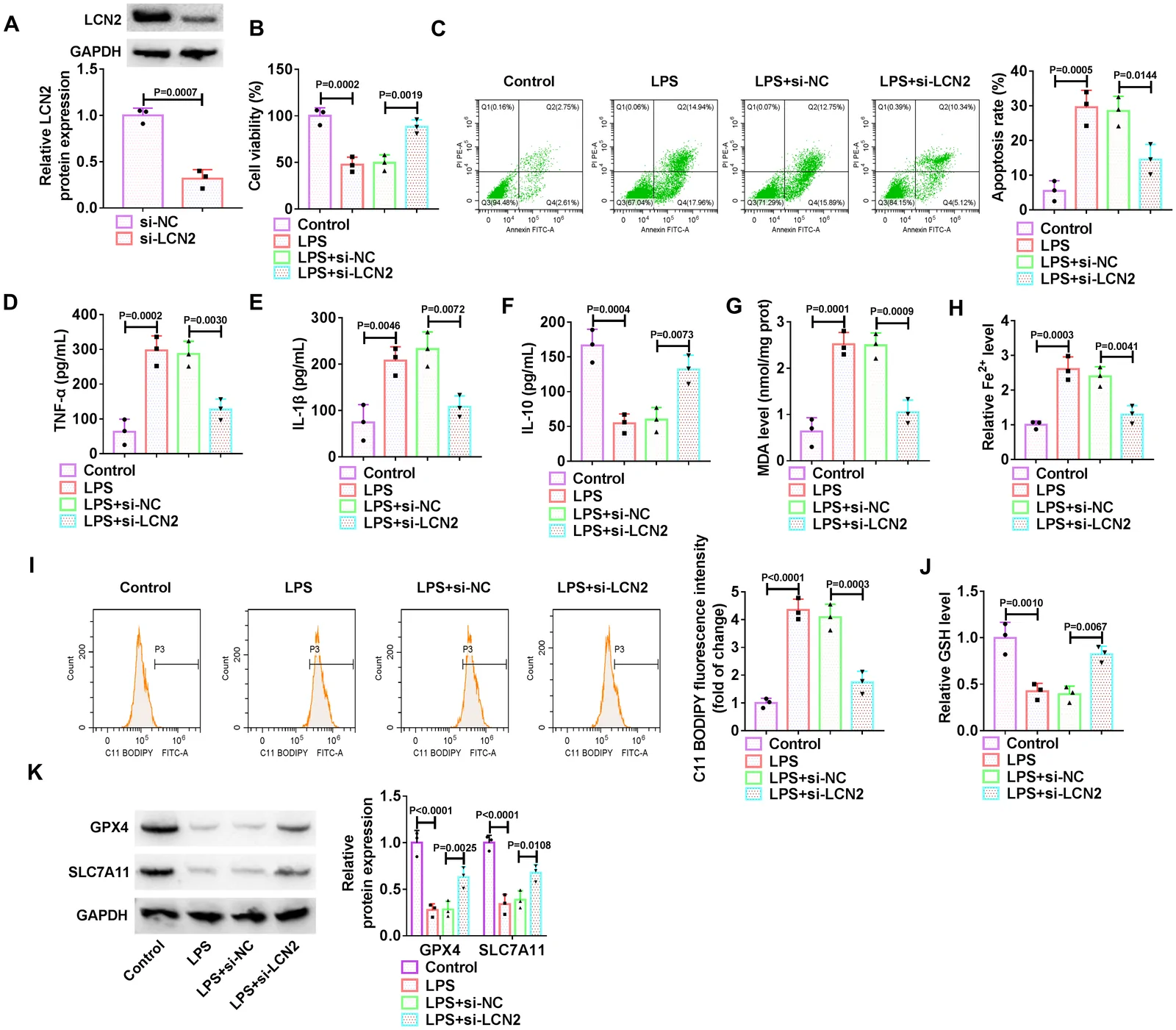
Effects of LCN2 silencing on LPS-induced endothelial dysfunction and ferroptosis *in vitro.* **(A)** LCN2 protein level was determined using western blot assay in HPMECs transfected with si-NC or si-LCN2, Unpaired *t*-test. **(B–K)** HPMECs were treated with 10 µg/mL LPS, 10 µg/mL LPS + si-NC, 10 µg/mL LPS + si-LCN2, or without (Control). **(B, C)** Cell viability and apoptosis were detected using CCK-8 and flow cytometry, one-way ANOVA. **(D–F)** Inflammatory cytokines (TNF-α, IL-1β, and IL-10) levels were measured using ELISA, one-way ANOVA. **(G, H)** MDA level and Fe^2+^ level were examined using corresponding detection kits, one-way ANOVA. **(I)** Lipid-ROS levels were analyzed using flow cytometer. **(J)** GSH level was measured using a commercial kit, one-way ANOVA. **(K)** GPX4 and SLC7A11 protein levels were determined using western blot assay, two-way ANOVA. n=3.

### FOXA1 transcriptionally activates LCN2 by binding to its promoter

To further identify the molecular mechanism of LCN2 in HPMECs, ChEA3 database and JASPAR website were applied to predict transcription factor binding sites. As a result, FOXA1 could bind to the LCN2 promoter region (**Figures 5A, B**). Moreover, western blot results presented that FOXA1 protein level was strikingly enhanced in LPS-treated HPMECs relative to the Control (**Figure 5C**). Besides, FOXA1 protein level was obviously reduced in si-FOXA1-transfected HPMECs versus cells with si-NC (**Figure 5D**), implying that the knockdown efficiency was successful. To assess whether FOXA1 affects the promoter activity of LCN2 in HPMECs, a dual-luciferase reporter assay was performed. As shown in **Figure 5E**, FOXA1 deficiency could prominently reduce the luciferase activity of cells expressing WT-LCN2 in HPMECs, but not the MUT-LCN2 group, indicating FOXA1 directly bound to the region of the LCN2 promoter. Meanwhile, the results of ChIP assay verified the interaction between FOXA1 and LCN2 at the promoter binding site (**Figure 5F**). In addition, LCN2 mRNA level was remarkably decreased after si-FOXA1 transfection in HPMECs (**Figure 5G**). Overall, these results implied that FOXA1 could directly bind to the promoter of LCN2 and promote its transcription in HPMECs.

**Figure 5:**
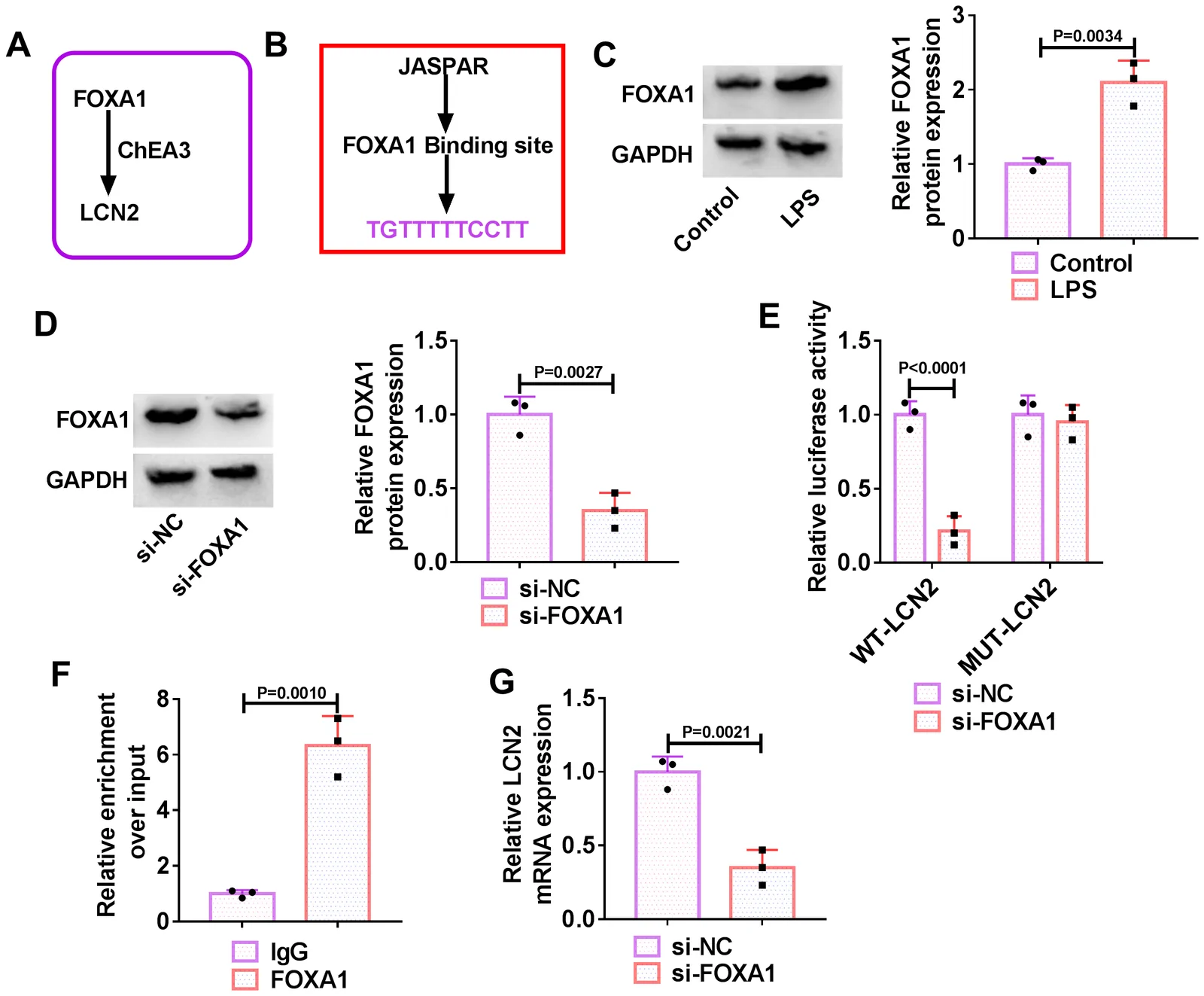
FOXA1 activated the transcription of LCN2. **(A)** The ChEA3 database predicts that the transcription factor FOXA1 is most likely to induce the activation of the transcription of LCN2. **(B)** The FOXA1 binding site on LCN2 promoter was analyzed using JASPAR website. **(C)** FOXA1 protein level was detected by western blot in HPMECs treated with 10 µg/mL LPS for 24 h or without (Control), Unpaired *t*-test. **(D)** Western blot analysis of FOXA1 protein level in HPMECs transfected with si-NC or si-FOXA1, Unpaired *t*-test. **(E, F)** Their interaction was verified using a dual-luciferase reporter assay (two-way ANOVA) and ChIP assay (Unpaired *t*-test) in HPMECs. **(G)** LCN2 mRNA level was detected by RT-qPCR assay in HPMECs transfected with si-NC or si-FOXA1, Unpaired *t*-test. n=3.

### FOXA1 knockdown attenuates LPS-induced HPMEC apoptosis, inflammation, and ferroptosis by regulating LCN2

Considering the regulatory role of FOXA1 in LCN2 expression in HPMECs, we further investigated whether the impact of FOXA1 on LPS-induced endothelial dysfunction was associated with LCN2. First of all, western blot results exhibited that LCN2 protein level was markedly increased in OE-LCN2-transfected HPMECs compared with the OE-NC group (**Figure 6A**). Functionally, FOXA1 deficiency could promote cell viability and reduce cell apoptosis in LPS-treated HPMECs, whereas these effects were partly overturned after OE-LCN2 co-transfection (**Figures 6B, C**). Beyond that, LPS exposure-induced inflammatory response in HPMECs was alleviated by FOXA1 downregulation, and this protection was partially overturned by OE-LCN2 co-transfection, as described by enhanced secretions of pro-inflammatory cytokines TNF-α and IL-1β (**Figures 6D, E**), and decreased anti-inflammatory cytokine IL-10 (**Figure 6F**). Regarding the characteristic phenotypes of ferroptosis, the lack of FOXA1 could markedly hinder MDA level (**Figure 6G**), intracellular Fe^2+^ level (**Figure 6H**), Lipid-ROS production (**Figure 6I**), increase GSH level (**Figure 6J**), and GPX4 and SLC7A11 protein levels (**Figure 6K**) in LPS-stimulated HPMECs, which were prominently reversed by LCN2 upregulation. All of these outcomes indicated that FOXA1 knockdown partly alleviated LPS-induced HPMEC dysfunction by targeting LCN2.

**Figure 6:**
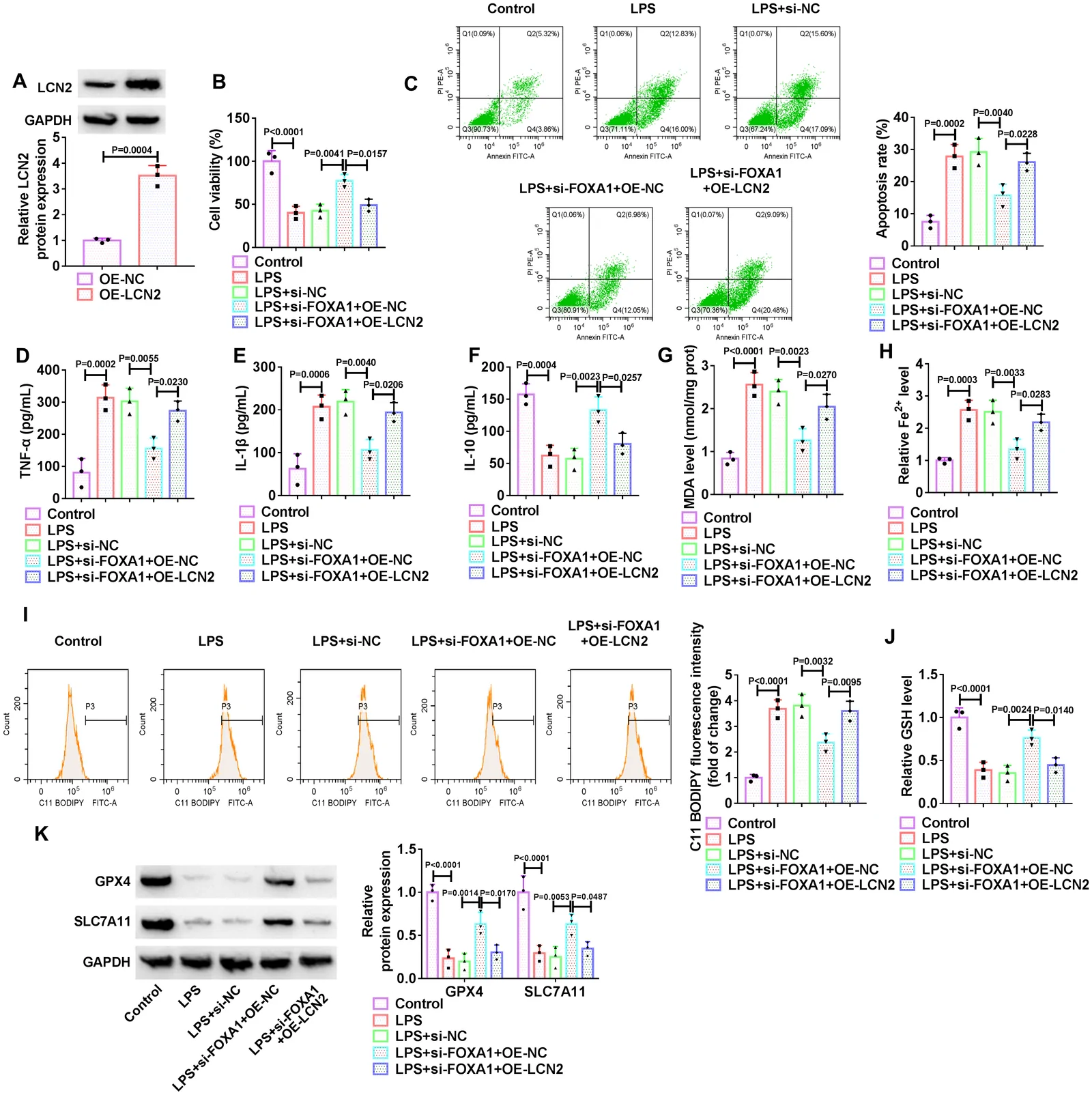
FOXA1/LCN2 affected LPS-induced HPMEC apoptosis, inflammation, and ferroptosis *in vitro.* **(A)** LCN2 protein level was detected using western blot assay in HPMECs transfected with OE-NC or OE-LCN2, Unpaired *t*-test. **(B–K)** HPMECs were treated with 10 µg/mL LPS, 10 µg/mL LPS + si-NC, 10 µg/mL LPS + si-FOXA1 + OE-NC, 10 µg/mL LPS + si-FOXA1 + OE-LCN2, or without (Control). **(B, C)** CCK-8 and flow cytometry were employed to determine cell viability and apoptosis, one-way ANOVA. **(D–F)** The concentrations of TNF-α, IL-1β, and IL-10 were examined using ELISA kits, one-way ANOVA. **(G, H)** MDA level and Fe^2+^ level were determined using corresponding detection kits, one-way ANOVA. **(I)** Flow cytometry was used to analyze Lipid-ROS level, one-way ANOVA. **(J)** GSH level was assessed using a commercial kit, one-way ANOVA. **(K)** Western blot assay was used to detect GPX4 and SLC7A11 protein levels, two-way ANOVA. n=3.

### TRIM9 promotes K48-linked polyubiquitination and degradation of LCN2

According to BioGRID database analysis, LCN2 was found to be associated with the ubiquitinating enzyme TRIM9 (**Figure 7A**). Furthermore, western blot assay displayed that TRIM9 protein level was remarkably decreased in LPS-treated HPMECs relative to the Control group (**Figure 7B**). The overexpression efficiency of TRIM9 was detected and presented in **Figure 7C**. Meanwhile, CoIP experiment verified the ability of endogenous TRIM9 to bind to LCN2 in HPMECs (**Figure 7D**). To further investigate the mechanism of LCN2 degradation thoroughly, a proteasome inhibitor (MG132) was added to HPMECs. Although high-expressed TRIM9 could reduce the protein level of LCN2 in HPMECs, this downregulation was reversed by the treatment of MG132 (**Figure 7E**). After that, we further asked whether TRIM9 exacerbates LCN2 degradation by performing a protein stability assay. After CHX (a chemical that inhibits protein synthesis) exposure, TRIM9 overexpression led to much faster degradation of LCN2 in HPMECs (**Figure 7F**). Since TRIM9 is a member of the TRIM family with E3 ligase activity, we sought to determine what type of polyubiquitination occurred in LCN2. So far, K48- and K63-linked chains have been confirmed as the most abundant and functionally well-characterized polyubiquitin chains. To determine which of them was presented in polyubiquitinated LCN2, HPMECs were co-transfected with OE-TRIM9 and K48 or K63 ubiquitin plasmids. After that, cells were subjected to an ubiquitination assay. As a result, it was found that when the amount of precipitated Flag-LCN2 was consistent, HA-Ub-K48, but not HA-Ub-K63, blocked TRIM9 overexpression-induced polyubiquitination of LCN2, (**Figure 7G**), suggesting that TRIM9 may promote K48-linked polyubiquitination and subsequent degradation of LCN2. Collectively, the above-mentioned data indicated that TRIM9 promoted K48-linked polyubiquitination degradation and subsequent of LCN2 in HPMECs.

**Figure 7:**
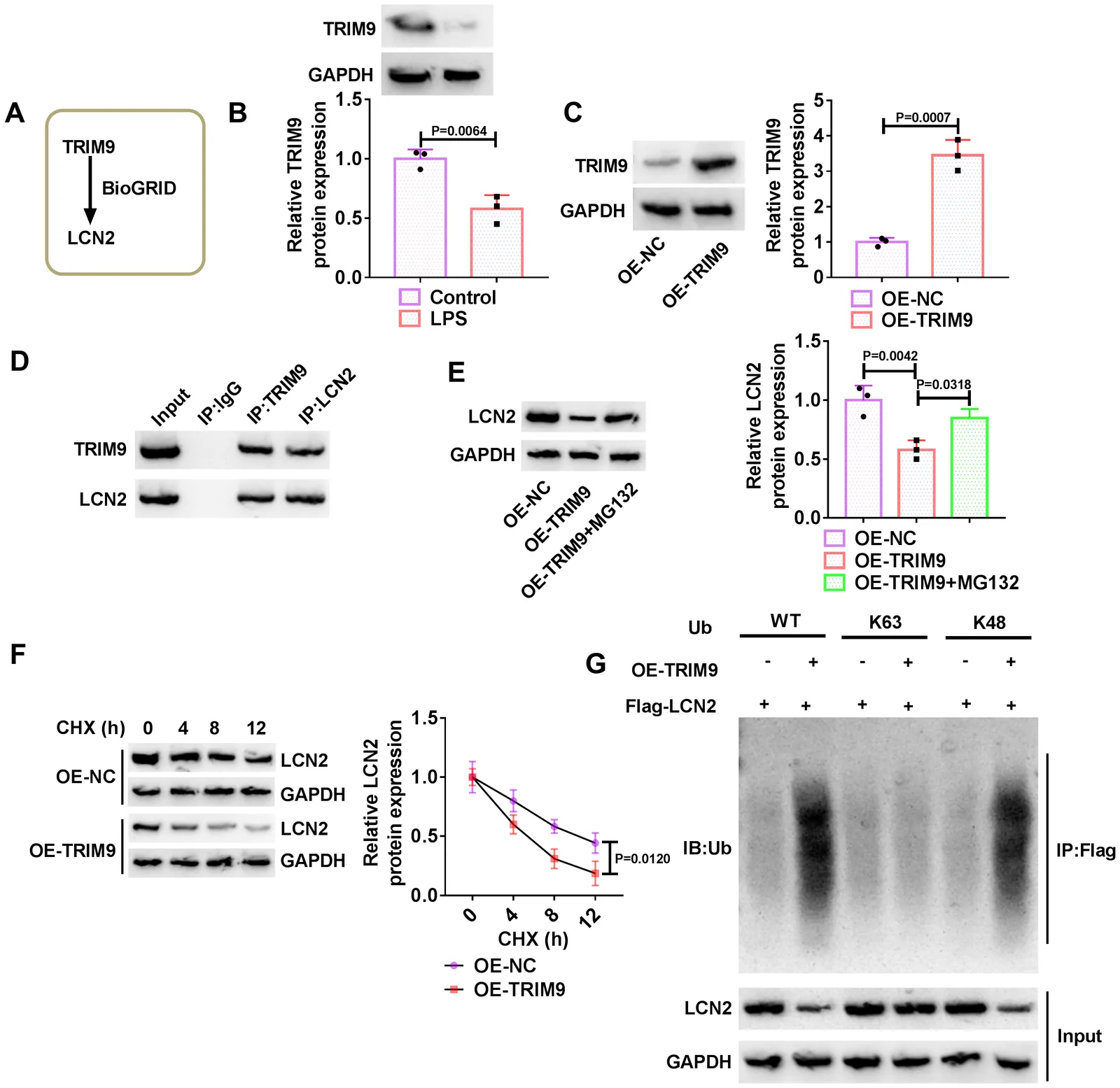
TRIM9 negatively regulates LCN2 expression through ubiquitination. **(A)** BioGRID database showed that the ubiquitinating enzyme TRIM9 can bind to LCN2. **(B)** TRIM9 protein level was assessed using western blot assay in HPMECs treated with 10 µg/mL LPS for 24 h or without (Control), Unpaired *t*-test. **(C)** Western blotting analysis of TRIM9 protein level in HPMECs transfected with OE-NC or OE-TRIM9, Unpaired *t*-test. **(D)** CoIP assay was performed to explore the interaction between endogenous TRIM9 and LCN2 in HPMECs, one-way ANOVA. **(E)** LCN2 protein level was detected using western blot assay in HPMECs treated with OE-NC, OE-TRIM9, or OE-TRIM9 + 10 µM MG132, two-way ANOVA. **(F)** Influences of TRIM9 upregulation on LCN2 protein stability after CHX treatment was assessed using western blot assay in HPMECs, one-way ANOVA. **(G)** Wild-type K48 and K63 Ub plasmids were co-transfected into HPMECs with or not with OE-TRIM9 or Flag-LCN2. Then, the type of polyubiquitination of LCN2 in HPMECs was determined using CoIP assay, one-way ANOVA. n=3.

### TRIM9 overexpression alleviates LPS-induced endothelial dysfunction *in vitro* by targeting LCN2

Additionally, to further determine whether TRIM9 could affect LPS-induced endothelial dysfunction by interacting with LCN2, rescue experiments were performed in LPS-treated HPMECs. After that, functional experiment analysis exhibited that enhanced cell viability and reduced cell apoptosis were observed due to the upregulation of TRIM9 in LPS-exposed HPMECs, while these effects were partly reversed after OE-LCN2 co-transfection (**Figures 8A, B**). In parallel, the protective effect of TRIM9 overexpression against LPS-caused cell inflammatory response and ferroptosis was effectively abrogated by LCN2 upregulation, accompanied by increased TNF-α and IL-1β (**Figures 8C, D**), decreased IL-10 (**Figure 8E**), higher MDA level, Fe^2+^ level, and ROS activity (**Figures 8F–H**), lower GSH level, and reduced GPX4 and SLC7A11 protein levels (**Figures 8I, J**). In addition, data showed that TRIM9 silencing repressed cell viability and increased ROS activity in LPS-treated HPMECs, which were partially reversed through LCN2 down-regulation (**Supplementary Figure S4**). Altogether, these findings indicated that LCN2 addition could partially weaken the repression of TRIM9 upregulation on LPS-induced HPMEC damage *in vitro*.

**Figure 8:**
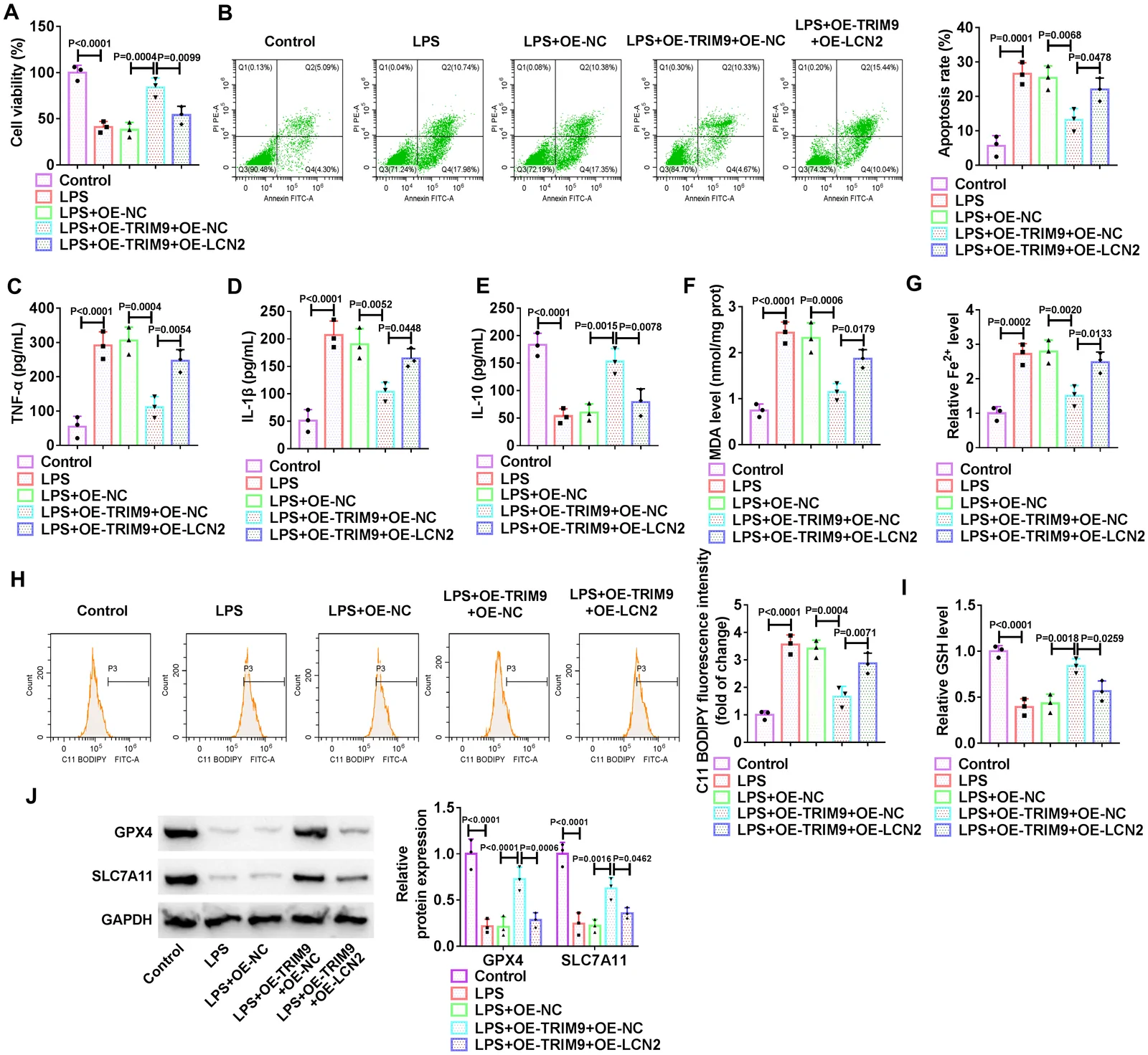
Effects of TRIM9 and LCN2 on LPS-induced endothelial dysfunction *in vitro.* HPMECs were treated with 10 µg/mL LPS, 10 µg/mL LPS + OE-NC, 10 µg/mL LPS + OE-TRIM9 + OE-NC, 10 µg/mL LPS + OE-TRIM9 + OE-LCN2, or without (Control). **(A, B)** Cell viability and apoptosis were detected using CCK-8 and flow cytometry, one-way ANOVA. **(C–E)** ELISA kits determined secretions of TNF-α, IL-1β, and IL-10, one-way ANOVA. **(F, G)** MDA level and Fe^2+^ level were measured using corresponding detection kits, one-way ANOVA. **(H)** Lipid-ROS levels were observed using flow cytometer, one-way ANOVA. **(I)** Special assay kits identified the level of GSH in HPMECs, one-way ANOVA. **(J)** GPX4 and SLC7A11 protein levels were measured using western blot assay, two-way ANOVA. n=3.

### LCN2 silencing attenuates lung injury in CLP rats

Based on cellular experimental findings, we hypothesized that LCN2 deficiency could alleviate lung injury *in vivo.* To further confirm the assumption, Ad-sh-LCN2 was constructed and injected into a CLP-induced septic rat model via the tail vein. Then, pathological observations of lung tissue were performed by HE and Masson staining (**Figure 9A**). In the sham group, HE staining revealed intact bronchial and alveolar architecture, thin and uniform alveolar septa, and patent alveolar spaces without inflammatory cell infiltration or exudation. In contrast, lung sections from CLP-induced septic rats exhibited hallmark features of acute lung injury, including marked thickening of the alveolar septa, diffuse interstitial and perivascular inflammatory cell infiltration (predominantly neutrophils and macrophages), alveolar collapse and consolidation, interstitial edema with protein-rich exudate filling the alveolar spaces, and scattered alveolar hemorrhage. Masson staining at 12 h post-CLP showed a mild increase in blue-stained extracellular matrix in the CLP group. It should be noted that this observation represents early, limited collagen accumulation or early matrix remodeling rather than established pulmonary fibrosis, as the 12-h time point corresponds to the acute phase of sepsis-induced lung injury. Notably, in the CLP+Ad-sh-LCN2 group, these pathological alterations were substantially attenuated, as evidenced by preserved alveolar architecture, reduced septal thickening, diminished inflammatory cell infiltration, less interstitial edema and exudation, and decreased collagen deposition compared with the CLP+Ad-sh-NC group. Quantitatively, the lung injury score was significantly reduced by LCN2 knockdown (**Figure 9B**), further supporting the histological observation that LCN2 silencing mitigates CLP-induced lung injury. Beyond that, LCN2 silencing could clearly mitigate CLP-caused increase in wet-dry mass ratio (W/D) of isolated lungs, implying that LCN2 deficiency could repress CLP-induced pulmonary edema (**Figure 9C**). In addition, ELISA analysis showed that CLP-triggered pro-inflammatory cytokines TNF-α and IL-1β enhancement and anti-inflammatory cytokine IL-10 were partly abolished by LCN2 downregulation (**Figures 9D–F**). Meanwhile, Fe^2+^ level was increased and GSH level was reduced in the CLP group, whereas these effects were markedly reversed through LCN2 silencing (**Figures 9G, H**). Besides, western blot analysis exhibited that CLP-mediated GPX4 (a star marker for ferroptosis) protein expression reduction and LCN2 level enhancement in lung tissues were obviously attenuated after Ad-sh-LCN2 introduction (**Figures 9I, J**). Therefore, these data suggested that LCN2 depletion could attenuate CLP-induced lung injury and inflammation, with accompanying alterations in ferroptosis-related markers *in vivo*.

**Figure 9:**
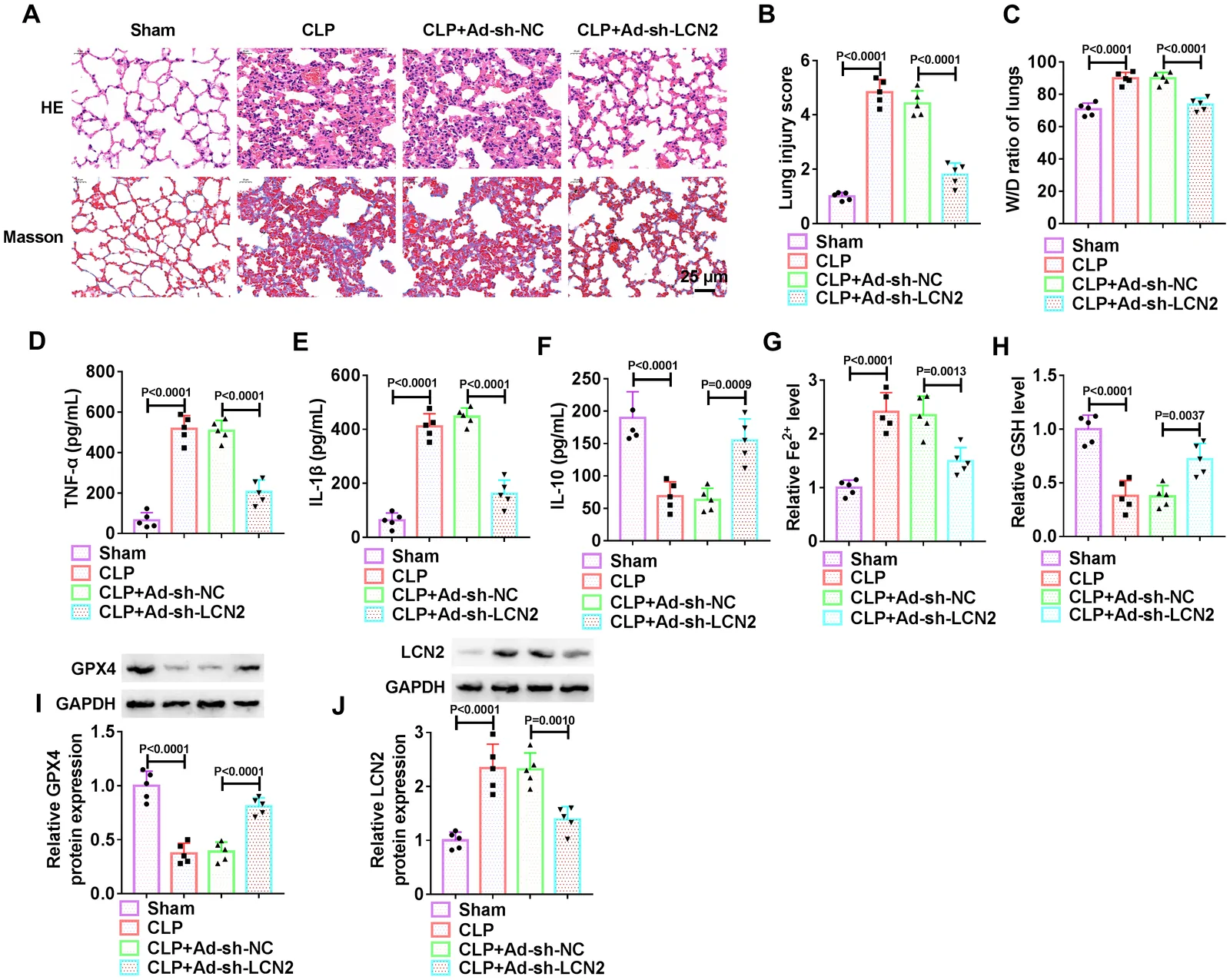
Deficiency of LCN2 could mitigate lung injury in CLP rats. **(A)** Pathological changes in rat lung tissues and lung fibers were determined by HE staining and Masson staining, Scale bar = 25 μm. **(B)** Tissue damage score of lungs. **(C)** W/D ratio was determined in rat lung tissues. **(D–F)** TNF-α, IL-1β, IL-10 secretions were assessed using ELISA kits in rat lung tissues. **(G, H)** Fe^2+^ level and GSH level were determined using corresponding detection kits. **(I, J)** GPX4 and LCN2 protein levels were detected using western blot assay in rat lung tissues. one-way ANOVA, n=3.

## Discussion

During the process of multiple organ dysfunction induced by sepsis, the lung is the earliest and most vulnerable target organ. At present, clinical studies have indicated that sepsis-induced ALI is closely correlated with increased mortality (34). Convincing evidence has indicated that sepsis-induced ALI arises from an excessive inflammatory response triggered by pathogenic microorganisms invading the body, accompanied by substantial ROS production. Of note, current research is progressively revealing the important effects of ferroptosis on the development of this complication (8, 35). Therefore, identifying suitable ferroptosis-related therapeutic targets for sepsis-induced ALI is crucial for related research.

Herein, to identify potential biomarkers and therapeutic targets, GSE32707 dataset was analyzed, yielding a total of 692 DEGs. Integrating WGCNA co-expression modules with ferroptosis-related genes from the GeneCards database, this study confirmed LCN2 as a potential ferroptosis-related candidate involved in sepsis-induced ALI progression. Moreover, the current work verified that LCN2 expression was increased in the LPS-treated HPMECs and in the lung tissues of the sepsis-induced rats. Furthermore, the silencing of LCN2 could relieve LPS-caused HPMEC inflammation and ferroptosis *in vitro*, and the lung damage in CLP-induced septic rats. These findings indicated that LCN2 could contribute to sepsis-induced ALI and is mechanistically linked to inflammatory response and ferroptosis, with the strongest mechanistic evidence derived from HPMECs and supportive evidence from the CLP model.

An important question arising from these findings is whether LCN2 upregulation in sepsis-induced ALI is pathogenic, compensatory, or context-dependent. Several lines of evidence suggest that LCN2 may play a context-dependent and potentially biphasic role. As a secreted acute-phase protein, LCN2 is rapidly induced under inflammatory and oxidative stress conditions, where it can sequester iron and limit bacterial growth, thereby exerting host-protective effects in certain infection settings (36, 37). However, in the context of sepsis-induced ALI, sustained LCN2 upregulation may become maladaptive. Herein, the present work validated that LCN2 could promote endothelial ferroptosis, inflammatory cytokine release, and barrier disruption, consistent with previous reports that LCN2 enhances macrophage iron accumulation and aggravates acute lung inflammation (38). In addition, the downregulation of LCN2 could alleviate sepsis-induced liver damage through repressing ferroptosis and decreasing oxidative stress (19), further supporting its pathogenic role in sepsis-related organ dysfunction. Notably, LCN2 has been described as playing paradoxical roles in lung inflammation—recruiting neutrophils and inducing pro-inflammatory signaling while also promoting anti-inflammatory M2-like macrophage polarization (39). These observations inferred that while the initial induction of LCN2 may represent a compensatory response to infection and iron overload, persistent and excessive LCN2 elevation may shift the balance toward a pathogenic role, exacerbating iron-dependent lipid peroxidation and tissue injury. This duality may explain why LCN2 has been reported to be both protective and detrimental in different disease contexts. Future studies using cell type-specific and time-controlled LCN2 manipulation will be necessary to determine the precise temporal and spatial window in which LCN2 transitions from a compensatory to a pathogenic mediator in sepsis-induced ALI.

Regarding the molecular mechanism, gene expression is typically directly controlled by transcription factors through binding to specific consensus sequences within enhancer or proximal promoter regions (20, 40). Herein, the present study identified FOXA1 as a transcription factor binding to the LCN2 promoter to activate its transcription. Located on chromosome 7q22.1, FOXA1 is a pioneer transcription factor that could initiate gene expression by binding to condensed chromatin and triggering the opening of local chromatin (21). Through specific DNA binding activity and DNA-bound transcriptional condensates, FOXA1 could bind to the promoter regions of target genes to influence their transcription, thus affecting sepsis progression. For example, the upregulation of FOXA1 could aggravate LPS-insulted vascular endothelial cell apoptosis and inflammation in sepsis by suppressing NRP2 transcription (41). Moreover, inhibition of FOXA1 could promote the transcription and expression of SLC7A11, thereby alleviating LPS-induced lung endothelial injury by suppressing ferroptosis (24). Consistent with these reports, FOXA1 expression was increased in LPS-treated HPMECs. Furthermore, functional experiments validated that the deficiency of FOXA1 could alleviate LPS-induced HPMEC apoptosis, inflammation, and ferroptosis by regulating LCN2 expression.

Extensive evidence has established that mRNA translation to proteins is a vital step in cellular gene expression, a process that undergoes stringent regulation in response to diverse extracellular stimuli and stress conditions. Among these mechanisms, the significance of ubiquitination modifications in translational regulation has become increasingly evident. This process precisely controls the balance between protein synthesis and degradation by tagging proteins with ubiquitin molecules (25, 42). Belonging to the ubiquitin E3 ligase family, TRIM proteins have been reported to partake in the modulation of ferroptosis and inflammatory response in various diseases (29, 43). Among them, TRIM9, a member of the TRIM family, is known for participating in ubiquitination and regulating protein metabolism (44, 45). However, the specific function of TRIM family members in sepsis-induced ALI remains incompletely understood. This study fills this research gap by demonstrating that TRIM9 influences sepsis-induced ALI through interaction with LCN2. Herein, the current work may suggest that TRIM9 promotes LCN2 degradation in association with increased K48-linked ubiquitination, resulting in a reduction in LCN2 protein levels. Moreover, rescue experiments validated that overexpressing LCN2 partly overturned the repression of TRIM9 upregulation on LPS-induced HPMEC injury and ferroptosis *in vitro.* In total, these observations preliminarily revealed a novel regulatory mechanism for LCN2 expression through FOXA1-activated transcription and TRIM9-mediated ubiquitination degradation, mechanistically validated *in vitro* and supported by *in vivo* CLP experiments. It is important to note that in our *in vivo* experiments, Ad-sh-LCN2 was administered 7 days prior to CLP induction, which represents a prophylactic intervention rather than a therapeutic one. This design was chosen to establish the causal contribution of LCN2 to the initiation and progression of sepsis-induced ALI by ensuring effective gene knockdown before the onset of septic insult. While this approach provides mechanistic insight into the role of LCN2 in disease pathogenesis, it does not directly address whether LCN2 inhibition could reverse established lung injury in a clinically relevant therapeutic setting. Given that patients with sepsis-induced ALI typically present after the onset of organ dysfunction, future studies employing LCN2 silencing or pharmacological inhibition after CLP induction (i.e., therapeutic intervention) will be essential to determine the translational potential of targeting LCN2 in established disease. Such studies, ideally combined with dose-response and time-window analyses, would provide a more comprehensive preclinical basis for developing LCN2-directed therapies for sepsis-induced ALI. It should be emphasized that the present findings are derived from *in vitro* and preclinical animal models, and therefore do not yet establish LCN2, FOXA1, or TRIM9 as clinically actionable therapeutic targets. Further validation in human samples and clinical studies will be required before any translational application can be considered.

Nevertheless, there are still some shortcomings in this research. First, this study did not performed clinical experiments, and more clinical tests need to be performed in the future. Second, although our data demonstrate that TRIM9 promotes K48-linked polyubiquitination and degradation of LCN2, the present study did not formally establish that the E3 ligase activity of TRIM9 is required for this effect. Overexpression of wild-type TRIM9 alone cannot exclude the possibility that TRIM9 acts as a scaffold or adaptor rather than through its enzymatic activity. To address this, future studies should employ catalytically inactive TRIM9 mutants (e.g., RING domain mutants with mutations in the zinc-coordinating cysteine/histidine residues, or a catalytically dead TRIM9 lacking E3 ligase activity) to determine whether TRIM9-mediated LCN2 ubiquitination and degradation depend on its E3 ligase activity. Third, the sample size of n = 5 per group, while consistent with previous studies in similar CLP models and supported by published guidelines for pilot animal experiments, may be insufficient to fully account for the substantial biological variability inherent to CLP-induced sepsis. Larger sample sizes determined by formal *a priori* power analysis will be necessary in future studies to strengthen the robustness of our conclusions. Fourth, while our *in vitro* experiments extensively validated the FOXA1–LCN2–TRIM9 regulatory axis, the *in vivo* experiments primarily focused on LCN2 knockdown and did not fully validate the complete axis in the septic lung. Demonstration of FOXA1 and/or TRIM9 manipulation *in vivo* (e.g., via adenovirus-mediated overexpression or knockdown of FOXA1/TRIM9 in CLP rats) would be necessary to establish that the proposed regulatory pathway operates in the intact septic lung rather than only in cultured HPMECs. In addition, the *in vivo* histological assessment was performed at 12 h after CLP, which corresponds to the acute phase of sepsis-induced lung injury. At this early time point, the mild increase in blue-stained extracellular matrix observed by Masson staining should not be equated with established pulmonary fibrosis or mature collagen deposition, but is more consistent with early, limited matrix remodeling. Longer time-course experiments with fibrosis-specific markers, such as collagen I/III, α-SMA, and hydroxyproline quantification, would be necessary to determine whether these early matrix changes progress to pulmonary fibrosis. Fifth, the LPS-induced HPMEC model used in this study represents a simplified *in vitro* system that cannot fully recapitulate the complex systemic and polymicrobial nature of clinical sepsis-induced ALI. LPS stimulation primarily activates TLR4-mediated signaling and induces endothelial inflammatory and cytotoxic responses, but does not reproduce the intricate interactions between endothelial cells and multiple immune cell populations (e.g., neutrophils, macrophages, and lymphocytes) or the polymicrobial infection characteristic of clinical sepsis. Therefore, our *in vitro* findings should be interpreted as mechanistic evidence for endothelial cell-intrinsic responses rather than a direct equivalent of clinical sepsis-induced ALI. Future studies employing more physiologically relevant models, such as CLP-induced sepsis *in vivo*, co-culture systems incorporating immune cells, or patient-derived samples, will be necessary to validate our findings in a more clinically relevant context. Sixth, although we separately validated the FOXA1→LCN2 transcriptional axis and the TRIM9→LCN2 ubiquitination axis, we did not perform an integrated epistasis experiment combining FOXA1 knockdown with TRIM9 manipulation (overexpression or knockdown) to determine whether these two regulators act independently or exhibit pathway interactions. Such an experiment—FOXA1 knockdown → TRIM9 manipulation → LCN2 expression → ferroptosis phenotype—would provide valuable insight into whether FOXA1 and TRIM9 independently regulate LCN2 or whether additional epistatic interactions exist. This integrated experimental design will be a key focus of our future investigation.

## Conclusion

In summary, these results provide *in vitro* mechanistic evidence that FOXA1 transcriptionally activates LCN2 by directly binding to its promoter region, thereby driving sepsis-induced ALI by increasing inflammation and ferroptosis. TRIM9 may promote K48-linked polyubiquitination of LCN2 and decrease its protein expression, thus forming a multi-level regulatory mechanism. In CLP rats, LCN2 knockdown attenuates lung injury and inflammation, with accompanying changes in ferroptosis-related markers, supporting the *in vivo* relevance of LCN2. These findings delineate a FOXA1–LCN2–TRIM9 regulatory axis that may govern ferroptosis and inflammation in experimental sepsis-induced ALI, and warrant further investigation before any translational application.

## Data Availability

The original contributions presented in the study are included in the article/**Supplementary Material**. Further inquiries can be directed to the corresponding author.

## Supplementary material

Supplementary Figure 1 The effects of LCN2, ferroptosis inhibitor (Ferrostatin-1), and ferroptosis activator (Erastin) on cell viability in LPS-treated HPMECs. (A) cell viability was assessed using CCK-8 assay in HPMECs treated with 10 µg/mL LPS, 10 µg/mL LPS + Ferrostatin-1 (ferroptosis inhibitor, 10 mM, 24 h, MedChemExpress), 10 µg/mL LPS + Erastin (ferroptosis activator, 5 mM, 24 h, MedChemExpress), or without (Control). (B) Cell viability was measured using CCK-8 assay in HPMECs treated with 10 µg/mL LPS, 10 µg/mL LPS + 10 mM Ferrostatin-1, 10 µg/mL LPS + 10 mM Ferrostatin-1 + OE-NC, 10 µg/mL LPS + 10 mM Ferrostatin-1+ OE-LCN2, or without (Control). (C) Cell viability was examined using CCK-8 assay in HPMECs treated with 10 µg/mL LPS, 10 µg/mL LPS + 10 mM Ferrostatin-1, 10 µg/mL LPS + 10 mM Ferrostatin-1 + si-NC, 10 µg/mL LPS + 10 mM Ferrostatin-1 + si-LCN2, or without (Control). n=3.

Supplementary Figure 2 Effects of si-LCN2 on tight junction proteins (Occludin and ZO-1) in LPS-treated HPMECs. Occludin and ZO-1 protein levels were determined using western blot assay in HPMECs treated with 10 µg/mL LPS, 10 µg/mL LPS + si-NC, 10 µg/mL LPS + si-LCN2, or without (Control). n=3.

Supplementary Figure 3 Effects of si-LCN2#2 knockdown on LPS-triggered endothelial dysfunction and ferroptosis *in vitro.* (A) LCN2 protein level was determined using western blot assay in HPMECs transfected with si-NC or si-LCN2#2. (B-I) HPMECs were treated with 10 µg/mL LPS, 10 µg/mL LPS + si-NC, 10 µg/mL LPS + si-LCN2, or without (Control). (B and C) Cell viability and apoptosis were assessed using CCK-8 and flow cytometry. (D-F) Inflammatory cytokines (TNF-α, IL-1β, and IL-10) levels were analyzed using ELISA. (G and H) MDA level and Fe^2+^ level were examined using corresponding detection kits. (I) Lipid-ROS levels were analyzed using flow cytometer. n=3.

Supplementary Figure 4 Effects of TRIM9 knockdown and LCN2 downregulation on LPS-mediated cell viability and Lipid-ROS *in vitro.* (A) TRIM9 protein level was assessed using western blot assay in HPMECs transfected with si-NC or si-TRIM9. (B and C) HPMECs were treated with 10 µg/mL LPS, 10 µg/mL LPS + si-NC, 10 µg/mL LPS + si-TRIM9 + si-NC, 10 µg/mL LPS + si-TRIM9 + si-LCN2, or without (Control). Then, cell viability and Lipid-ROS level were detected using CCK-8 assay and flow cytometry. n=3.
