# Supplementary material for: FOXA1-driven transcription and TRIM9-mediated ubiquitination of LCN2 modulate ferroptosis in experimental sepsis-induced acute lung injury
Source: Front Immunol. 2026 Oct 1;17:1822078. doi: 10.3389/fimmu.2026.1822078 (PMC13668516; doi:10.3389/fimmu.2026.1822078)

The original western blots of Fig3

C

Repeat 1 **Repeat 2** **Repeat 3**

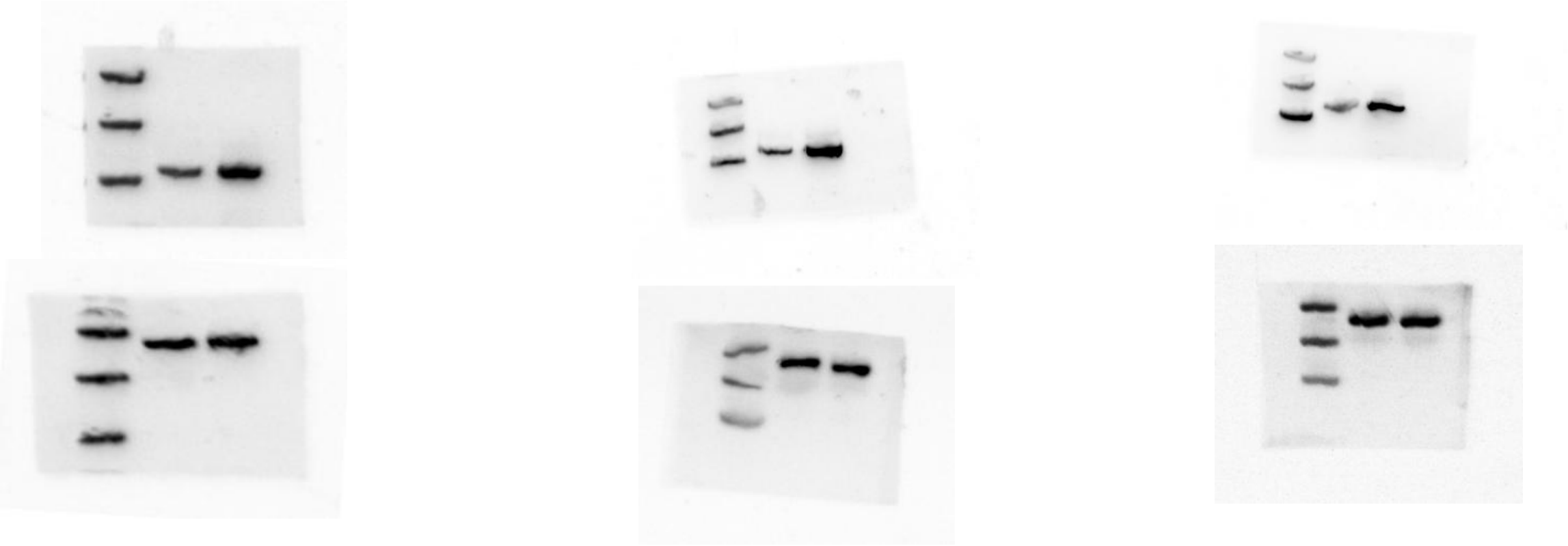

LCN2

GAPDH

The original western blots of Fig4

A

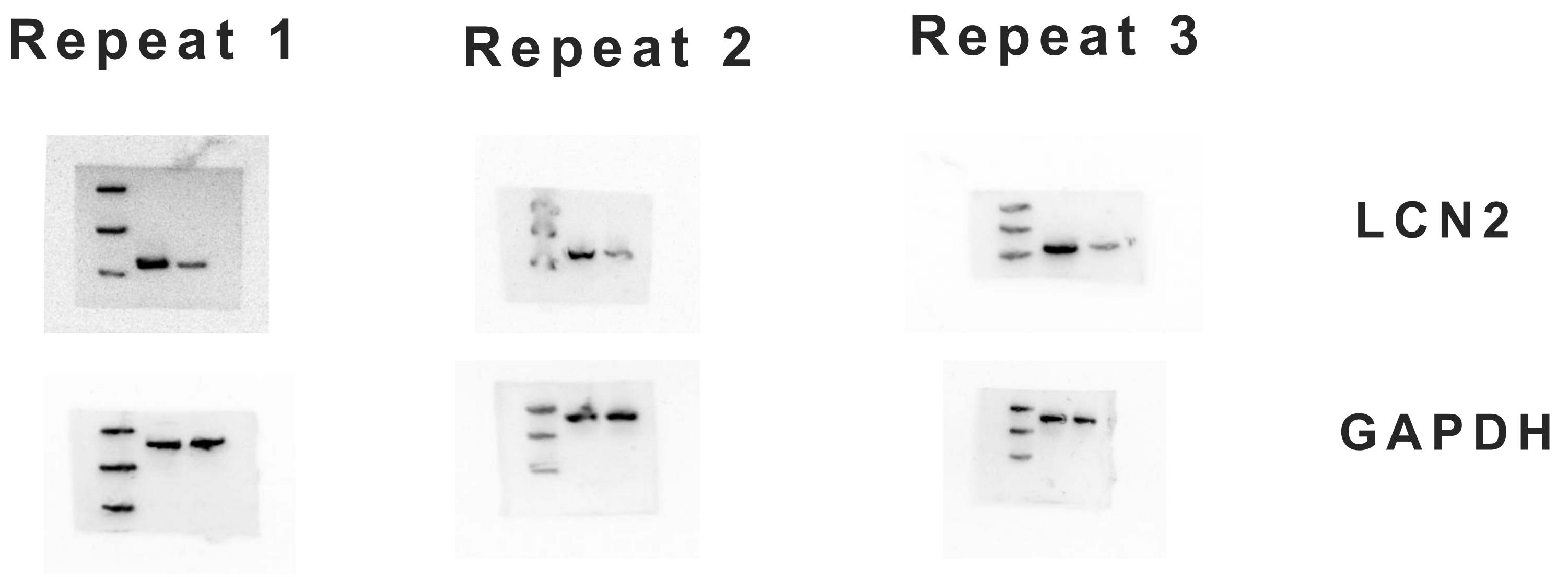

The original western blots of Fig5

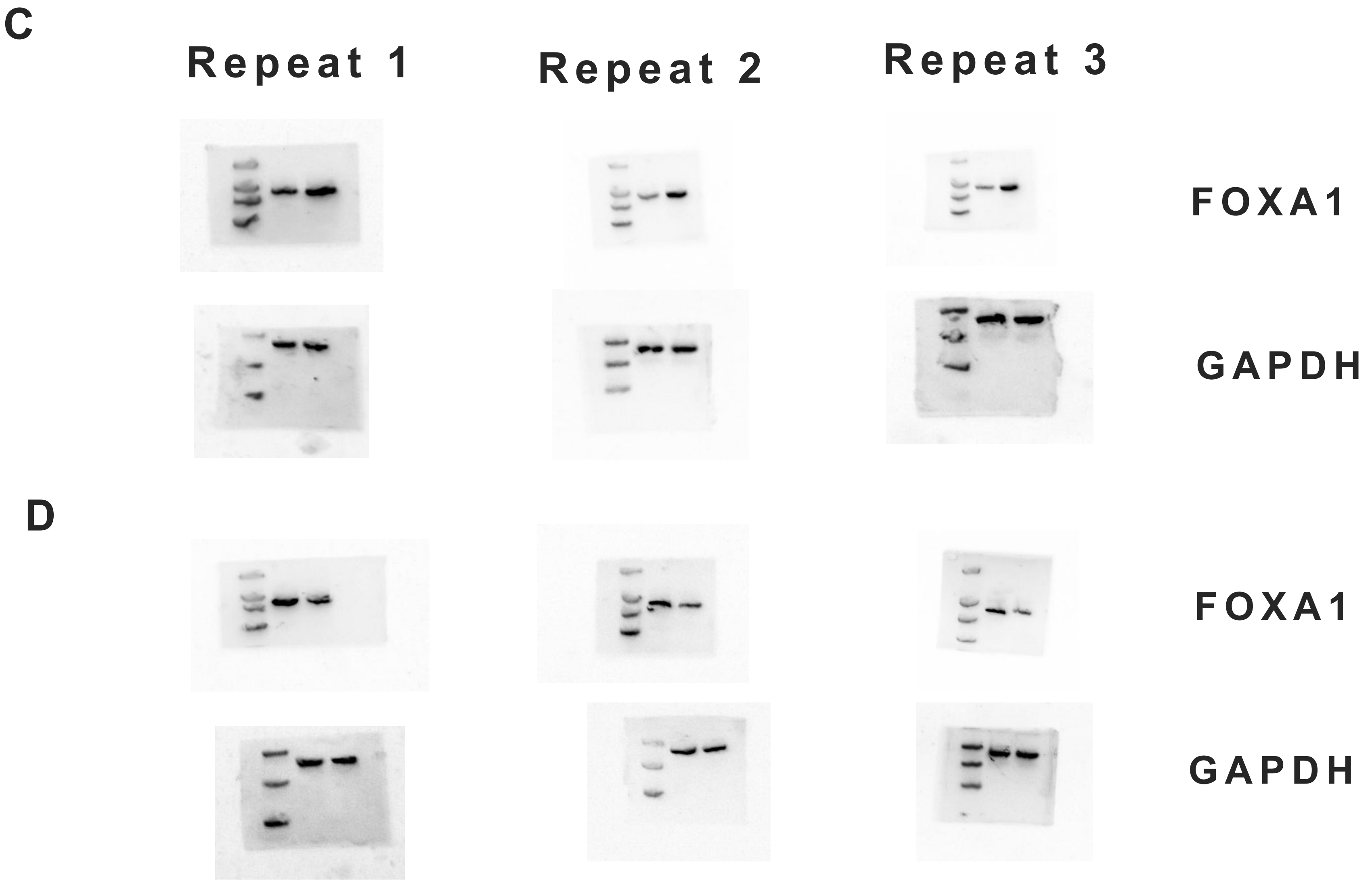

The original western blots of Fig6

A

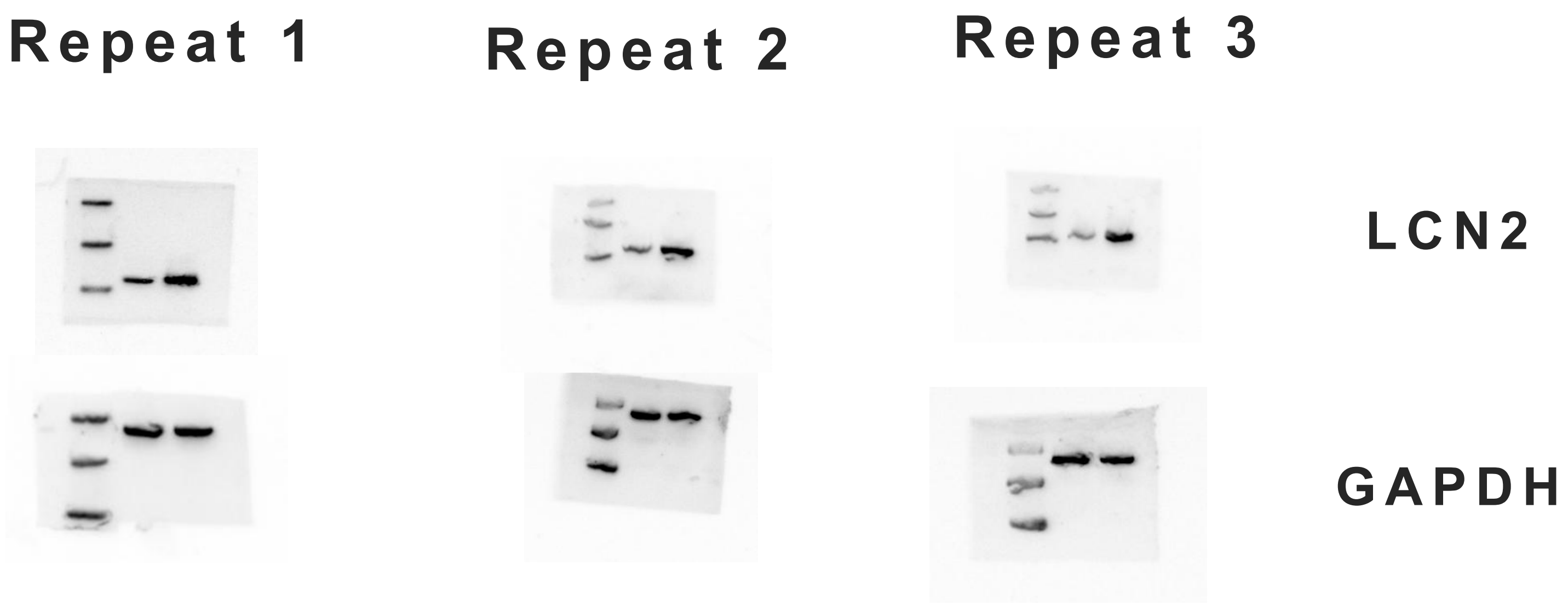

The original western blots of Fig7

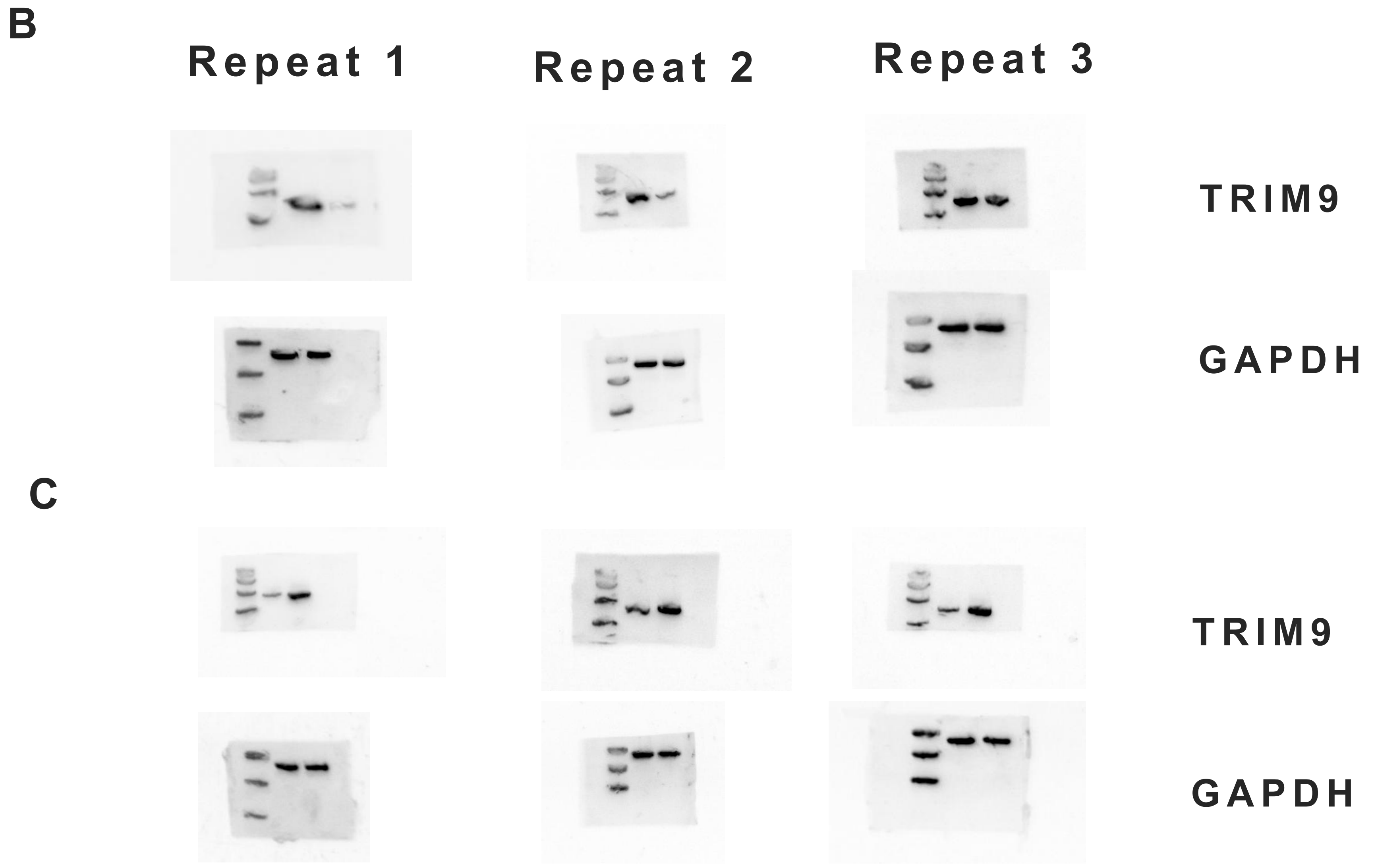

The original western blots of Fig7

D

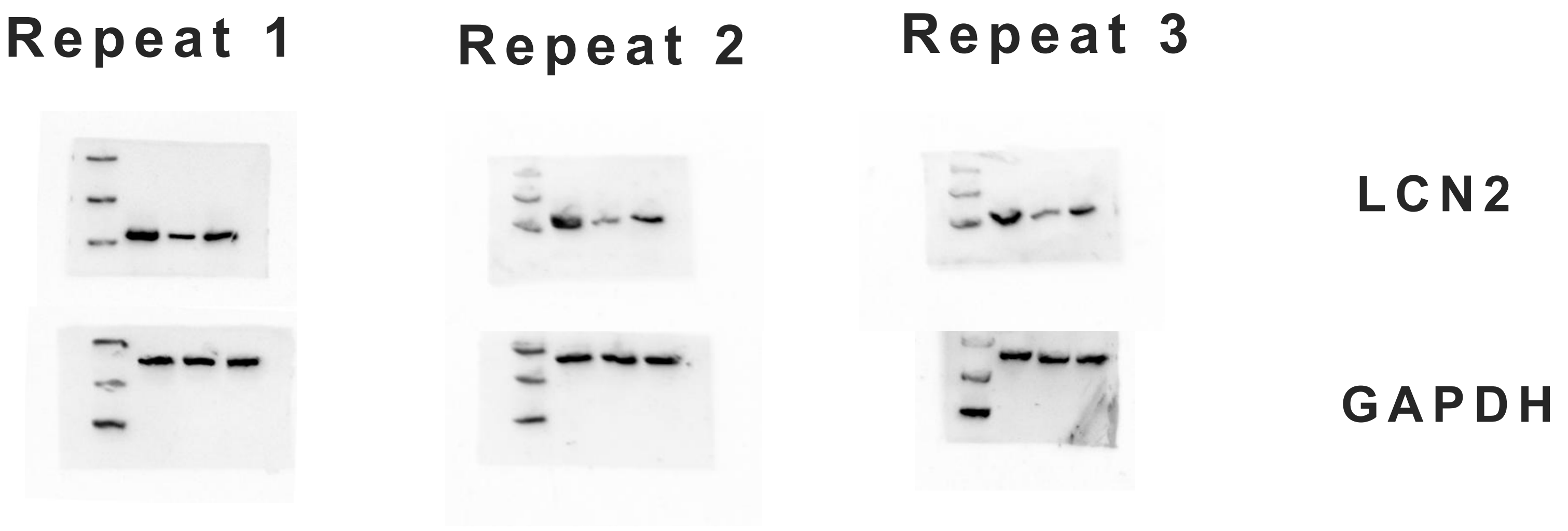

E

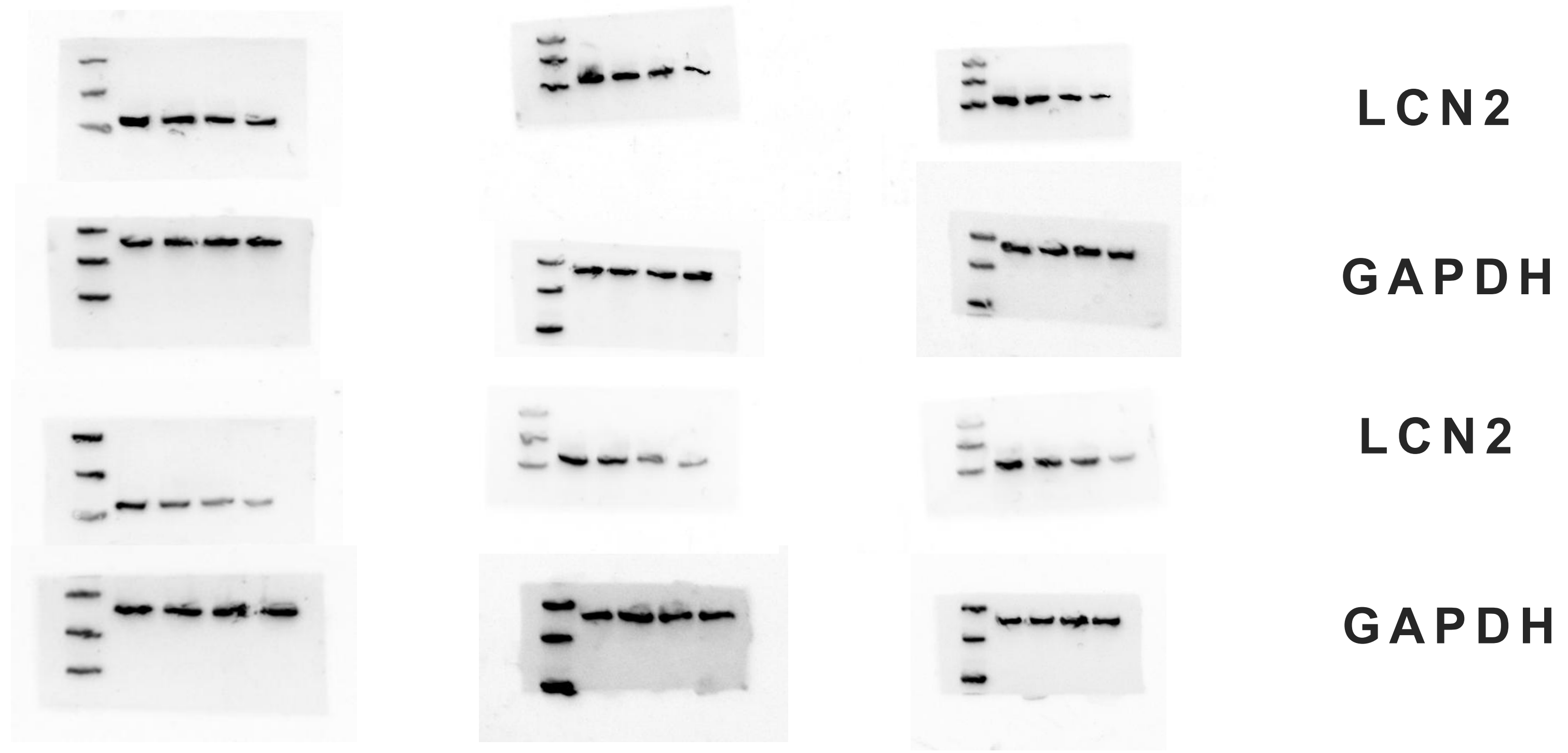

The original western blots of Fig7

F

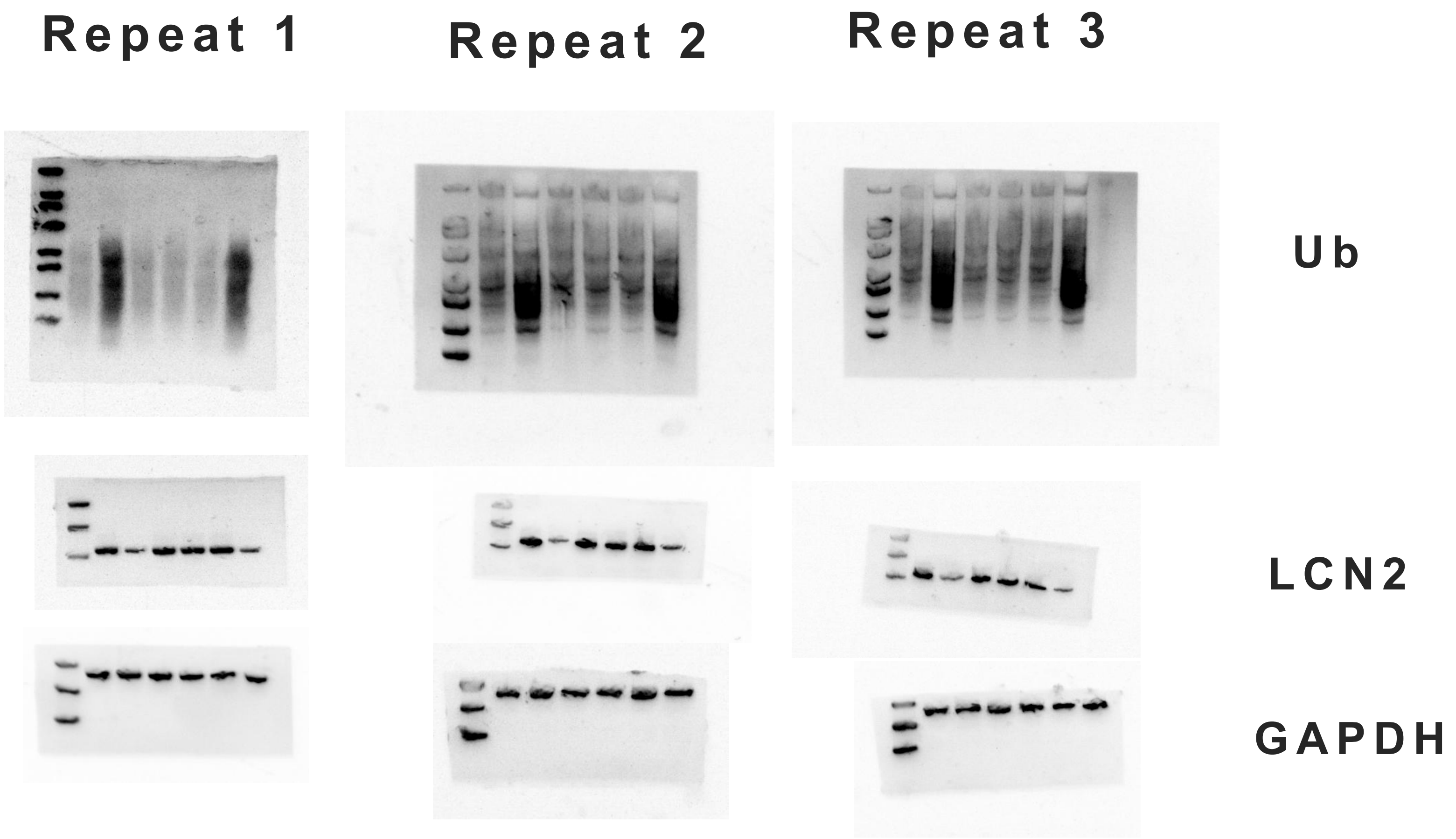

The original western blots of Fig9

G

Repeat 1 Repeat 2 Repeat 3

GPX4

GAPDH

H

LCN2

GAPDH

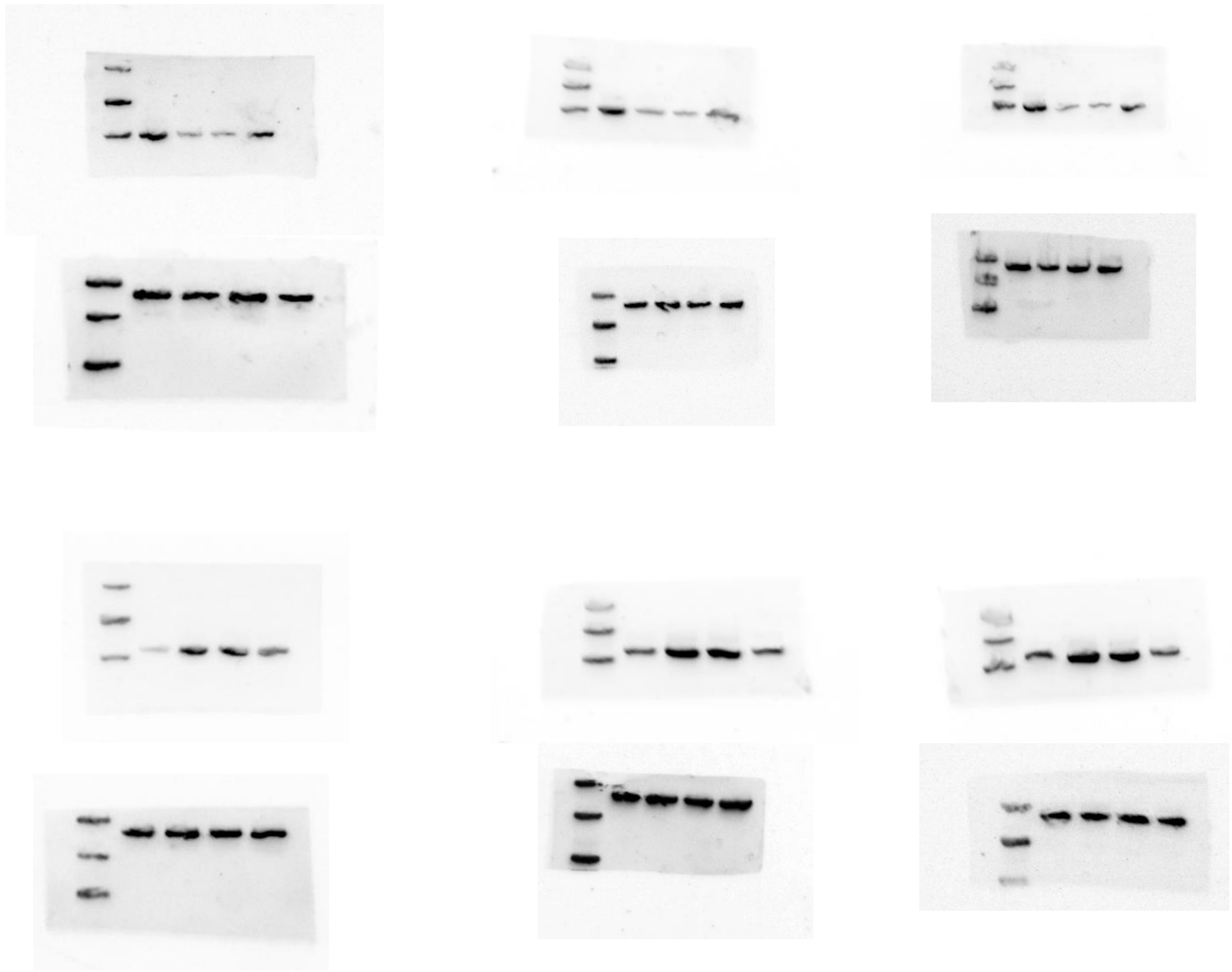

Supplement: Supplementary file 1 [file DataSheet1.pdf]
